# Construction of nanomaterials as contrast agents or probes for glioma imaging

**DOI:** 10.1186/s12951-021-00866-9

**Published:** 2021-05-03

**Authors:** Wei Zhao, Xiangrong Yu, Shaojun Peng, Yu Luo, Jingchao Li, Ligong Lu

**Affiliations:** 1 Zhuhai Precision Medical Center, Zhuhai Interventional Medical Center, Zhuhai People’s Hospital (Affiliated With Jinan University), Zhuhai, 519000 Guangdong China; 2 School of Chemical Science and Engineering, Tongji University, 1239 Siping Road, Shanghai, China; 3 College of Chemistry, Chemical Engineering and Biotechnology, Donghua University, Shanghai, 201620 China

**Keywords:** Glioma imaging, Nanomaterials, Blood–brain-barrier, Contrast agents, Probes, Biomedical imaging

## Abstract

Malignant glioma remains incurable largely due to the aggressive and infiltrative nature, as well as the existence of blood–brain-barrier (BBB). Precise diagnosis of glioma, which aims to accurately delineate the tumor boundary for guiding surgical resection and provide reliable feedback of the therapeutic outcomes, is the critical step for successful treatment. Numerous imaging modalities have been developed for the efficient diagnosis of tumors from structural or functional aspects. However, the presence of BBB largely hampers the entrance of contrast agents (Cas) or probes into the brain, rendering the imaging performance highly compromised. The development of nanomaterials provides promising strategies for constructing nano-sized Cas or probes for accurate imaging of glioma owing to the BBB crossing ability and other unique advantages of nanomaterials, such as high loading capacity and stimuli-responsive properties. In this review, the recent progress of nanomaterials applied in single modal imaging modality and multimodal imaging for a comprehensive diagnosis is thoroughly summarized. Finally, the prospects and challenges are offered with the hope for its better development.

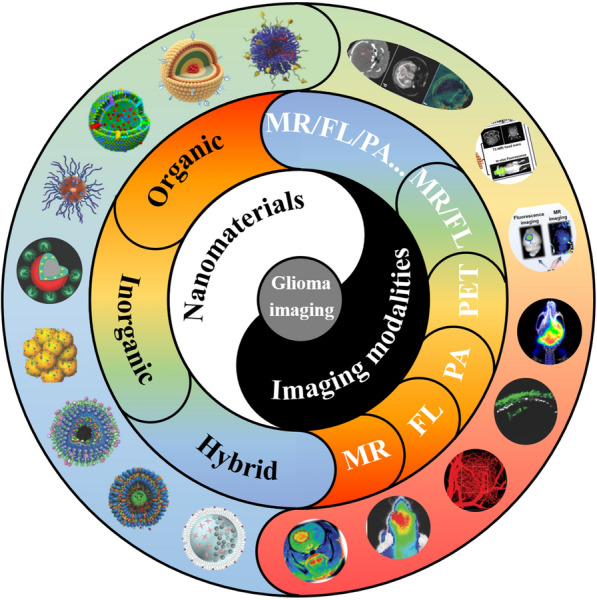

## Introduction

Primary brain tumors, which originate from the brain, are one of the most common cancers among children, adolescents, and young adults (ages below 39) [1]. Specifically, they are the leading cause of cancer-related death in the 0–14 age group, outpacing even leukemia according to a recent report [2]. Gliomas, which are evolved from normal stromal (glial) cells, account for nearly 50% of the primary brain tumors, thus largely threatening human health. According to the classification proposed by World Health Organization (WHO, 2016), gliomas can be divided into diffuse gliomas (divided into three subtypes: IDH, IDH mutant 1p/19-non-codeleted, IDH mutant 1p/19-codeleted), and nondiffuse gliomas (divided into four subtypes: other astrocytic tumors, ependymal tumors, other gliomas, mixed neuronal-glial tumors). Glioma in IDH mutant 1p/19-non-codeleted and IDH mutant 1p/19-codeleted types are also termed glioblastoma (GBM), the most common malignant brain tumor which is characterized by high aggressiveness, mortality, recurrence, and poor prognosis, with the median survival of only 15–16 months even aftercare treatment [3–5].

Currently, the standard therapeutic regimen includes surgical resection followed by chemo/radiotherapy. Although significant progress has been made, malignant gliomas remain incurable, mainly attributed to the following reasons: (i) the heterogeneous and infiltrative nature of GBM, which obscures the boundary between normal and abnormal tissues, leading to incomplete excision of the neoplastic region and future recurrence; (ii) the existence of blood–brain-barrier (BBB), which strictly restricts the entry of drugs, contrast agents (Cas), and probes into the brain. As the important biological barrier, BBB is comprised of, from the inside-out, cerebral endothelial cells (ECs) with a tight junction, surrounding base-membrane, pericytes, and the endfeet of astrocytes. It plays an essential role in maintaining the homeostasis of the central nervous system (CNS), protecting the brain from the contamination of neurotoxic substances. However, on the other hand, the tight junction and absence of fenestrations on ECs contribute to the very limited permeability of functional molecules, of which this situation is further aggravated by the high-level expression of ATP-binding cassette (ABC) transporters like P-glycoprotein closely related to drug efflux [6–8]. As a result, more than 98% of small-molecule drugs, and almost all large molecules are prevented from entering into the brain [9].

Precise diagnosis of gliomas is the first vital procedure to efficiently improve the treatment outcomes, which largely depends on the development of imaging technology. A variety of imaging modalities, such as magnetic resonance imaging (MRI), computed tomography (CT) imaging, fluorescence imaging (FLI), nuclear medical imaging, and photoacoustic imaging (PAI), can provide pathological information from different aspects. For example, traditional MRI (T_1_-weighted or T_2_-weighted) and CT reflect the anatomical changes of neoplastic tissues while nuclear medical imaging reveals the metabolic changes at the molecular level, as a kind of functional imaging modality. In most cases, contrast agents (Cas) or probes are needed for improving the contrast for better distinguishing the region of interest, which is critical to tumor boundary delineation for guiding surgical resection as well as tracing the therapy outcomes. Unfortunately, the existence of BBB seriously impedes the performance of Cas, leading to incomplete excision and poor prognosis in significant measure. Therefore, it’s imperative to search for effective methods to traverse the BBB.

As technologies advance, considerable amounts of strategies have been developed to overcome the difficulties brought by BBB, of which the mechanisms can be categorized as follows: receptor-mediated transcytosis (RMT: peptides and proteins) [10, 11], adsorption-mediated transcytosis [12] (AMT), cell-mediated penetration [13], cellular barrier (lack of pinocytosis and bulk flow transcytosis), simple diffusion (CO_2_, O_2_, alcohol, lipophilic drugs < 400 Da and8 hydrogen bonds), CMT (carbohydrates, fatty, acids, monocarboxylic acids, amino acids, hormones, vitamins, organic anions and cations, and nucleotides) [14], major facilitators (ω_3_ fatty acids), ions and water (Na^+^/H^+^; Cl^−^/HCO_3_^−^; Na^+^/K^+^/2Cl^−^; Na^+^/Ca^2+^), active efflux (ABC transporters, drugs, xenobiotic products, and drug conjugates), clearance of neurotoxic substances, diffusion of molecules across brain ECS and another physical or chemical process, such as cavitation effect from high intensity focused ultrasound (HIFU) [15, 16]. Nanomaterials, with improved in vivo behaviors compared to small molecules, such as prolonged blood circulation time and enhanced accumulation in tumor sites, have been extensively explored to serve as nanoprobes for tumor imaging and therapy in the past decades [17–20]. The abundance of functional groups on the surface allows the conjugation of targeting molecules for RMT-mediated BBB crossing. Also, other BBB traversing strategies can be combined with nanoprobes for better imaging performance: magnetic guidance combined with nanobubble-assisted focused ultrasound (FUS) exposure to disrupt the BBB may be another strategy to (i) deliver multimodality imaging contrast agents (for both diagnostic ultrasound (US) and magnetic resonance imaging), (ii) catalyze safe BBB disruption, and (iii) deliver drugs via carriers for efficacious therapy of brain disease treatments [21]. Besides, the high loading capacity of nanocarriers permits the co-existence of two or more kinds of contrast agents, thus realizing multimodal imaging for a comprehensive diagnosis. Furthermore, large varieties of nanomaterials can act as Cas themselves, such as Fe_3_O_4_, MnO_2_, Au nanorods, semiconducting polymers, which greatly extended the range of Cas [22–26].

**Scheme 1:**
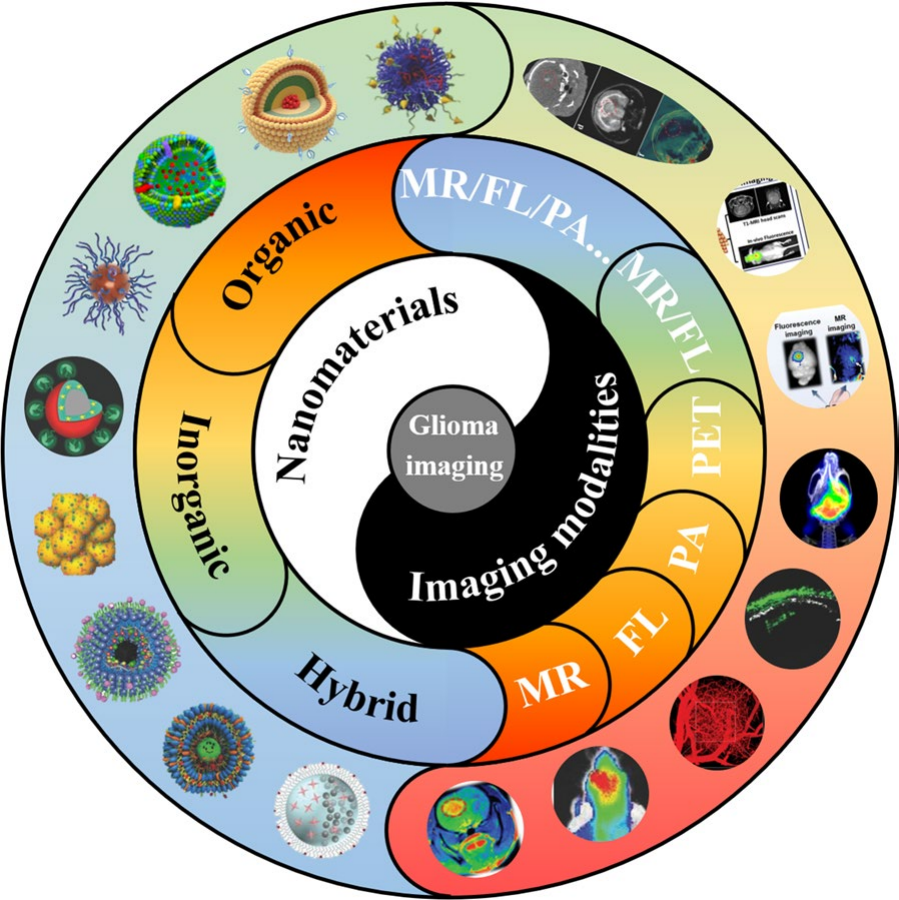
Schematic illustration of the nanomaterials applied for glioma imaging. Generally, those kinds of nanomaterials can be divided into organic nanoparticles such as liposomes and synthetic polymers, inorganic nanoparticles like Au and SiO_2_ nanoparticles, and organic/inorganic hybrid nanoparticles such as Au nanorods encapsulated by metal–organic frameworks (MOFs). A large variety of imaging modalities, including magnetic resonance (MR) imaging, fluorescence (FL) imaging, photoacoustic (PA) imaging, positron emission computed tomography (PET), can be achieved by nanomaterials for comprehensive detection and diagnosis of glioma. Nanomaterials are endowed with blood–brain-barrier (BBB) penetration ability through surface modification of targeting molecules, the assistance of external stimuli such as focused ultrasound, or other strategies (Reprint with permission [38, 65, 148]. Copyright 2018, Wiley–VCH. Reprint with permission [49, 104, 170, 207]. Copyright 2020, Wiley–VCH. Reprint with permission [105, 161]. Copyright 2019, American Chemical Society. Reprint with permission [32, 66, 198, 205]. Copyright 2017, Wiley–VCH. Reprint with permission [106, 139]. Copyright 2016, Wiley–VCH. Reprint with permission [209]. Copyright 2019, Wiley–VCH. Reprint with permission [171]. Copyright 2019, Elsevier)

In this review, the recent progress of nanomaterials in glioma imaging is summarized, with several sections presented according to different imaging modalities, as shown in Scheme 1. In each chapter, the basic introduction of the corresponding imaging modality is firstly given, followed by a detailed summary of the nanomaterials application. The mechanisms of materials synthesis and BBB penetration are also involved. Finally, we offer our perspectives on the prospects and challenges in this field. It is expected that nanomaterials can play more and more important roles in glioma imaging, ultimately benefiting the patients.

## MRI

MRI is extensively used for disease detection clinically due to its high spatial resolution, unlimited tissue penetration, negligible radiation damage. Especially, MRI is intrinsically endowed with an excellent anatomical resolution to soft tissues, which makes it a potent imaging modality for brain-related diseases. such as glioma. Cas is usually needed in MRI as they can alter the relaxation time of the surrounding protons, rendering the location of interest more distinguishable [27, 28]. The high degree of invasiveness of glioma during the growth process leads to intertwining with normal tissues and nerves around the tumor, and the outline is blurred. At present, the most commonly used magnetic resonance contrast agents are small-molecule contrast agents based on gadolinium ions, which lack specific identification of tumor tissues and normal tissues around tumors, and the contrast is not high. Therefore, based on the intersection of materials, chemistry, biomedicine, and other disciplines, the design, and construction of contrast agents or molecular probes that highly identify tumor tissues and normal brain tissues and nerves around tumors has great potential for clearly delineating the boundaries of gliomas. Herein, the recent progress of nanomaterials for different MRI modalities in glioma detection is reviewed.

### Nanomaterials for T_1_-weighted MRI

T_1_-W MRI is most frequently adopted in the clinic for glioma diagnosis by distinguishing the varied spin–lattice (longitudinal, T_1_) relaxation time of different neurological tissues. It reflects the recovery extent of the longitudinal magnetization intensity of the protons after the excitation of a radio-frequency pulse. Cas for T_1_-W MRI can efficiently reduce the longitudinal relaxation time of the ambient protons, therefore generating a brighter signal compared to the places with no Cas.

**Fig. 1:**
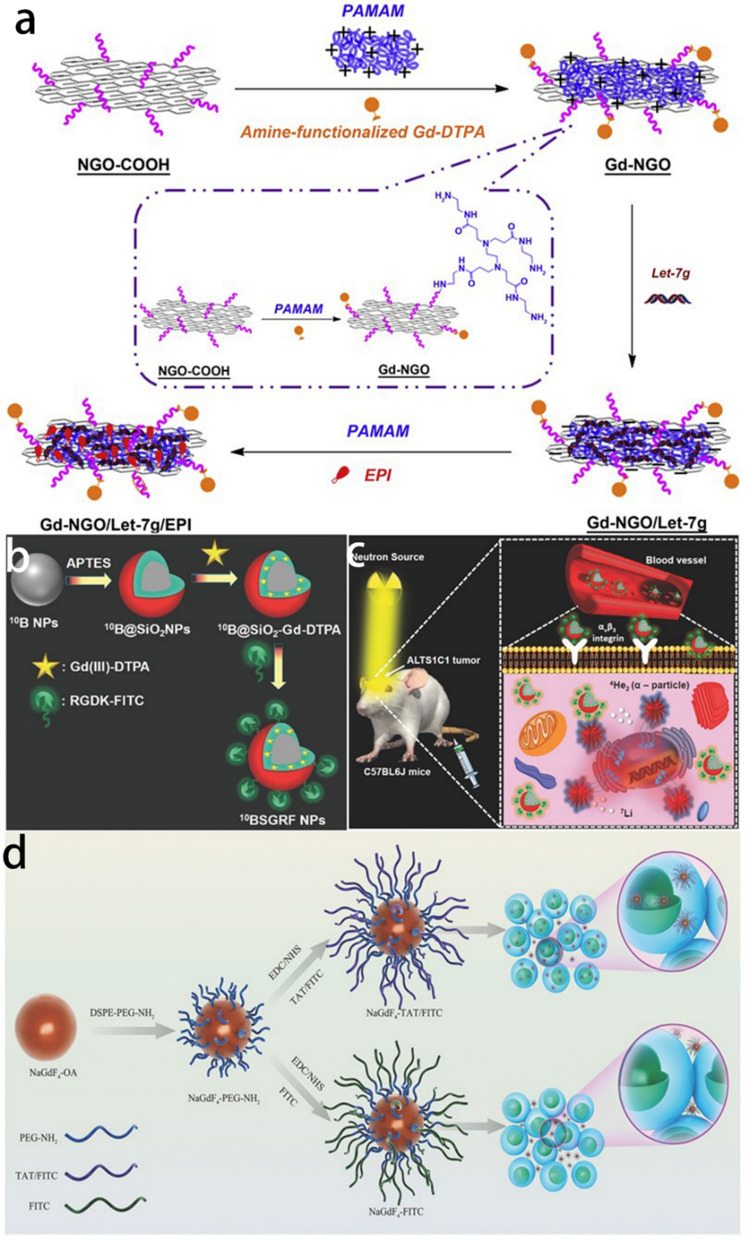
**a** Scheme of the procedure for the preparation of Gd-NGO/Let-7g/EPI. Reprint with permission [29]. Copyright 2014 Elsevier. **b** Schematic representation for the synthesis of the 10BSGRF NPs. **c** Schematic representation of the in vivo MR imaging-guided targeted boron neutron capture therapy using 10BSGRF NPs. Reprint with permission [32]. Copyright 2017, WILEY–VCH. **d** The process of synthesis of cell-penetrating NaGdF4-TAT/FITC, non-cell-penetrating NaGdF4-FITC, and their labeling on adoptive T-cells. Reprint with permission [38]. Copyright 2018, WILEY–VCH

Immobilization of Gd-based small organic molecules onto the surface or into the cavity of nanocarriers is a feasible and facile strategy that combines the advantages of each component. The nanocarriers can be nano-graphene oxide [29], Au nanoparticles [30], polymers [31], SiO_2_ [32]. For example, Yang et al*.* synthesized a kind of nanocomposite where ethylenediamine modified Gd-DTPA is covalently bound to the nanographene oxide [29]. Interestingly, the longitudinal relaxation rate (r_1_) is much higher than that of Gd-DTPA alone, which is likely ascribed to the increased molecular reorientation time (τ_R_) originating from the strong interaction between Gd ions and graphene oxide [33, 34]. This phenomenon implies a simple way to boost the r_1_ value of the nanoprobes, which favors improving the sensitivity of MRI (Fig. 1a). In another work, Gd-DTPA was coupled with SiO_2_ shell through electrostatic interactions where ^10^B nanoparticles were encapsulated inside to conduct Boron neutron capture therapy. Cyclic RGD peptide was modified on the surface of the nanoplatform for BBB crossing. Consequently, the brain tumor region was efficiently brightened for the pre-therapy diagnosis and post-therapy evaluation (Fig. 1b, c) [32].

Besides Gd-based small molecules, another category involves a range of inorganic nanoparticles usually containing Gd, Fe, or Mn elements. Gd_2_O_3_ is the typical member of Gd-involved inorganic Cas which has been widely studied for glioma imaging [35–37]. In this research, Gd_2_O_3_ nanoparticles were obtained through a “polyol-like” or thermal decomposition method, which generally led to ultra-small size (sub 10 nm) that benefited from improving the specific surface area. More Gd ions were exposed on the surface for interacting with the surrounding water molecules and therefore, a much higher r_1_ value was derived. Poly (ethylene glycol) (PEG), SiO_2,_ or other biocompatible polymers are often decorated onto the surface of Gd_2_O_3_ to regulate the in vivo behaviors. Moreover, the targeting moiety, like cyclic RGD or chlorotoxin. is another vital part of efficiently traversing BBB. Apart from Gd_2_O_3_, it has also been reported that NaGdF_4_ nanoparticles (~ 3 nm) with high r_1_ value (8.93 mM^−1^ s^−1^) were utilized for labeling adoptive T lymphocyte to achieve the MRI monitoring of the migration of T-cells towards glioma region during the relevant immunotherapy process [38]. TAT, a kind of HIV-1 transactivator peptide, was modified onto the hydrophilic NaGdF_4_ for boosting affinity with adoptive T cells (Fig. 1d). Gadolinium metallofullerene, where the Gd-contained compound is encapsulated inside the cage structure of fullerene, is also studied for its potential to act as T_1_-W MRI Cas [39, 40]. Fillmore et al*.* successfully synthesized carboxyl functionalized Gd_3_N@C_80_ to conjugate with targeting peptide, interleukin-13 (IL-13), for specific imaging of glioma cells in vitro. Utilizing convection-enhanced delivery (CED), effective glioma imaging was achieved [39]. However, owing to the invasive nature of CED which may impair the normal brain, as well as the negatively charged surface of the nanoplatform, there was still much room for improvement. Li et al*.* further ameliorated this Gd-based Cas through amino modification which gave the positive surface charge. Consequently, effective orthotopic glioma imaging was realized by intravenous injection [40]. Interestingly, Fe_3_O_4_ nanoparticles, which are widely used as T_2_-W MRI Cas, also possess the latent capacity for T_1_-W MRI. Luo et al*.* fabricated ultra-small Fe_3_O_4_ with an average diameter of 2.7 nm through the solvothermal method [41]. cRGD-modification imparted the targeting ability and as a result, effective imaging of the subcutaneous glioma model was achieved. Besides, Gd_2_O_3_ and Fe_3_O_4_ can be combined for MRI as well as Fenton-reaction mediated glioma therapy [42].

**Fig. 2:**
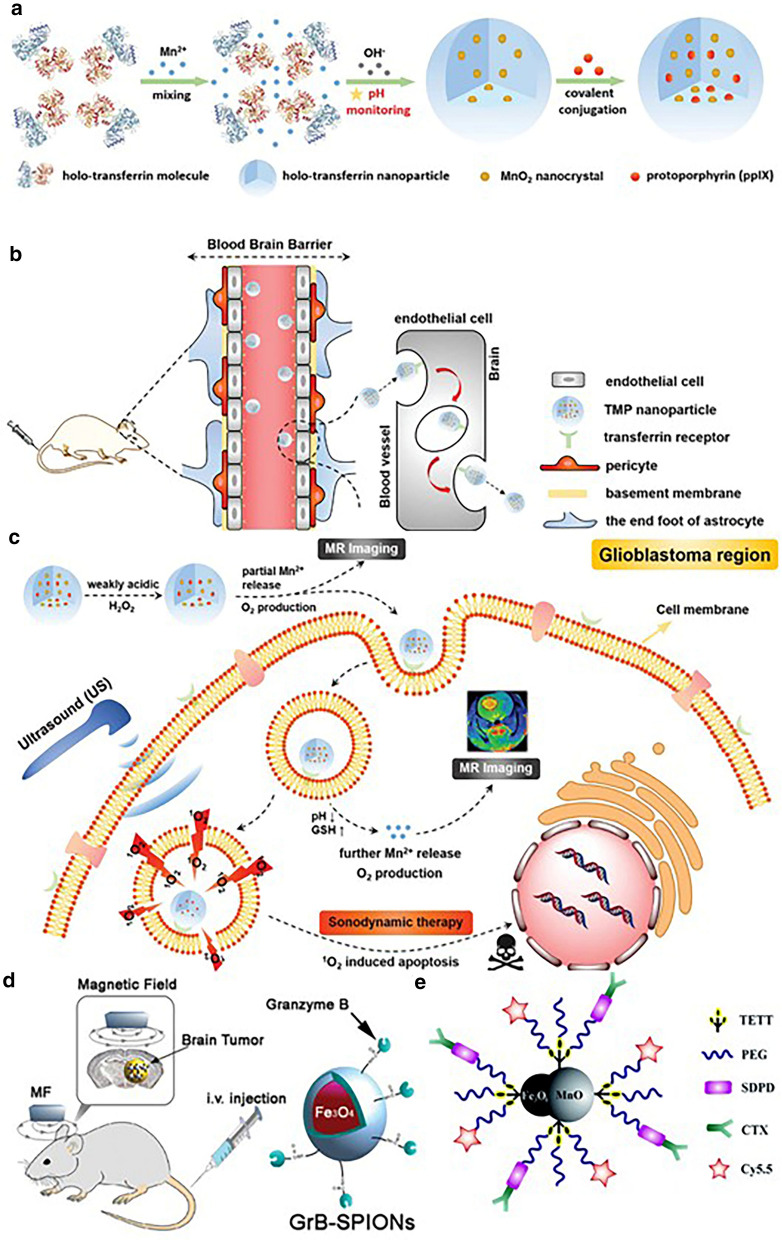
**a** The synthesis route of TMP. **b** TMP nanoparticles crossing BBB through binding to transferrin receptors over-expressed on endothelial cells. **c** TMP nanoparticles wielding the function as both MR contrast agents and sono-sensitizers after surmounting BBB to achieve precise theranostics of glioblastoma. Reprint with permission [49]. Copyright 2020, WILEY–VCH. **d** Scheme of the GrB-SPIONs and the mechanism of action. Reprint with permission [60]. Copyright 2020, WILEY–VCH. **e** Schematic illustration of Fe_3_O_4_/MnO–Cy5.5–CTX NPs (Reprint with permission [82]. Copyright 2015, Royal Society of Chemistry)

The afore-mentioned Cas have one thing in common: they all feature “always-on” MRI signals in the course of detection. Specific glioma imaging is obtained relying on the conjugated targeting moieties. However, because a variety of proteins in the blood circulatory system will combine with nanoparticles to form a protein corona, which shields the targeting molecules connected to the nanoparticles from specifically recognizing tumor cells. Meanwhile, there are hydrolytic enzymes in the blood that will attack the covalent bond between the targeted molecule and the nanoparticle, causing the targeted molecule to fall off and lose its targeting function [43]. Therefore, it is expected that the self-enhancing contrast agent generated in response to the MR signal generated by using nanoparticles to identify the tumor-microenvironment factor between the tumor tissue and the normal tissue without the need for targeting molecules is highly desired. Manganese oxides, which decompose to release paramagnetic Mn ions triggered by tumor-microenvironment (weakly acidic, excessive H_2_O_2,_ and GSH), are explored for glioma imaging. Generally, Mn oxides are formed on the biocompatible macromolecules that act as the growing template, such as poly(allylamine hydrochloride) (PAH) [44], hyaluronic acid (HA) [45], human or bovine serum albumin (HSA/BSA) [46–48] and transferrin (Tf) [49] through a redox reaction or biomineralization process. Fu et al. synthesized HA-MnO_2_ nanoparticles through directly mixing NaMnO_4_ with HA aqueous solution wherein HA served as both reductant and template for MnO_2_ growth [45]. As a result, a high r_1_ value (13.93 mM^−1^ s^−1^) under simulated tumor microenvironment, compared to the much lower r_1_ value of 1.59 mM^−1^ s^−1^ under physiological conditions, demonstrated the superiority of Mn oxides. Following intravenous injection, HA-MnO_2_ nanoparticles could effectively brighten the glioma region for 3 days. Nevertheless, the direct oxidation of HA would inevitably destroy its structure, leading to the loss of the inherent targeting ability of HA. Therefore, an improved biomineralization method was proposed by Chen’s group in which holo-transferrin (holo-Tf) was exploited as the growing template [49] (Fig. 2a). By finely regulating the solution pH during the addition of the NaOH solution for MnO_2_ nucleation, the structural integrity of holo-Tf was well preserved, which guaranteed the homing capacity of holo-Tf. Inspiringly, the synthesized nanocomposite with a responsively drastic improvement of r_1_ value (11.07 mM^−1^ s^−1^ compared to 0.78 mM^−1^ s^−1^ in normal physiological conditions) could potentially traverse BBB and target the glioma region, with its boundary clearly demarcated (Fig. 2b, c). In addition, other kinds of Mn oxides, like MnOx and MnO, have also been reported for targeted glioma imaging [48, 50].

### Nanomaterials for T_2_-weighted MRI

T_2_-weighted MRI is another basic MR imaging modality wherein the spin–spin (transverse) relaxation time (T_2_) of protons is differentiated. Because of the long T_2_ of water molecules, water-rich pathological tissues, such as glioma-related edema or infarction, produce bright signals under the T_2_-W scanning model. T_2_-W CAs, mostly Fe_3_O_4_ nanoparticles, are used to decrease T_2_ of the surrounding tissues, thereby generating a dark signal in comparison with the Cas-free areas. Fe_3_O_4_ nanoparticles used for Cas are superparamagnetic (SPION), usually divided into two categories according to their size (> 50 nm or < 20 nm), of which the latter is also called ultra-small superparamagnetic iron oxide (USPIO). The transverse relaxivity (r_2_) of such materials can reach up to several hundred mM^−1^ s^−1^.

Classically, Fe_3_O_4_ nanoparticles are coated with a biocompatible layer, such as PEG, and further conjugated with targeting moieties for specific T_2_-weighted MRI of glioma [51–54]. Kawamura et al*.* studied how the density of conjugated targeting peptide, cRGD, influenced the accumulation efficiency at the tumor neovasculature [55]. Results showed that a 40% substitution of the distal site of PEG-coated on the synthesized polyion complex vehicles (PICs) achieved the optimal outcome, which inspired the researchers to further load SPION inside the nanocarrier for the glioma diagnosis. As mentioned above, SPION could also be encapsulated inside or modified onto the nanocarriers to improve the stability and prolong the blood circulation time [56–59]. As regards targeting moieties, in addition to conventional targeting ability, some grafted molecules can induce the apoptosis of tumor cells simultaneously. For example, Shevtsov and his co-workers designed a kind of SiO_2_ shell coated SPION, on which granzyme B was grafted [60]. As an effector molecule excreted by T cells and natural killer (NK) cells, granzyme B has a high affinity towards, and selectivity for Heat shock protein 70 (Hsp 70), which is over-expressed on diverse tumor types including glioma but remains moderate in normal cells. After being internalized, apoptosis in a perforin-induced manner is triggered to eliminate tumor cells (Fig. 2d). Apart from synthetic coating materials like PEG, PEI, or SiO_2_, natural existing nanocarriers such as exosomes and cell membranes, have also been reported to wrap SPION followed by conjugation of targeting peptides for glioma imaging [61]. Moreover, owing to the superparamagnetic property of Fe_3_O_4_ for T_2_-W MRI, the targeting efficiency can also be enhanced using a magnet as an accessorial method [60, 62, 63], and the therapy outcomes can be ameliorated through an alternating magnetic field (AMF)-induced hyperthermia. Interestingly, not only can the coating layers around SPION be natural, but also the SPION itself can be synthesized in situ in living organisms. Boucher et al*.* managed to obtain RGD-modified magnetosomes comprised of biomineralized Fe_3_O_4_ nanocrystals coated by biological membranes that were generated inside magnetotactic bacteria under genetic editing technology [64]. The derived magnetosomes exhibited uniform size distribution and ultra-high r_2_ (560 ± 35 mM^−1^ s^−1^) value, favoring targeted glioma imaging. This work paves a new way for the fabrication of Fe-based MRI contrast agents for glioma detection. In addition, SPIO NPs are also reported to cooperate with chemotherapeutic drugs or siRNA to construct traceable nanoplatform for real-time monitoring of glioma therapy outcomes [65, 66].

Although ferumoxytol has not been approved by the Food and Drug Administration (FDA) as an imaging contrast agent, off-label use of the agent has shown benefit in patients with contraindications to gadolinium-enhanced MRI [67, 68]. Hoffman et al*.* have conducted clinical trials on 12 patients with primary or secondary brain malignancies to determine the optimum time of delayed contrast enhancement of ferumoxytol, and to compare ferumoxytol and gadolinium contrast agents for magnetic resonance angiography and perfusion. The results showed that the maximal ferumoxytol enhancement intensity was at 24 to 28 h after administration, and the enhancing volume subsequently expanded with time into a non-gadolinium-enhancing, high T_2_-weighted signal region of the tumor-infiltrated brain. Dynamic studies were assessed with both agents, indicating early vascular leak with gadolinium but not with ferumoxytol [69]. Hamilton and his co-worker’s study display that intracranial metastatic disease detection with ferumoxytol-enhanced MRI was not inferior to detection with gadolinium-enhanced MRI. Ferumoxytol-enhanced MRI could improve workup and monitoring of patients with brain metastases if gadolinium-enhanced MRI is contraindicated [70].

### Nanomaterials for T_1_–T_2_ dual-modal MRI

Although T_2_-weighted MRI features relatively higher sensitivity compared to T_1_-weighted MRI, the generated dark signal can sometimes be confused with other pathological conditions such as bleeding, blood clots, calcification, or metal deposits, leading to lowered accuracy for diagnosis. What’s worse, the high susceptibility of SPIO distorts the surrounding magnetic field, which usually accounts for the blurred images. However, from another perspective, the superparamagnetic property in the alternating magnetic field contributes to magneto-thermal conversion through the Néel–Brownian relaxation process, thus generating hyperthermia for demolishing malignancies. Taken together, it’s very promising to develop nano-sized Cas with T_1_–T_2_ dual-modal imaging ability that integrates the superiorities of each mode [71, 72].

Classically, the preparation of this kind of Cas includes four strategies: (i) direct conjugation of T_1_-weighted Cas with T_2_-weighted Cas [73, 74]; (ii) element doping (e.g., Mn or Gd-doped Fe_3_O_4_ nanoparticles) [75–77]; (iii) proper regulation of magnetic nanoparticles, like size and magnetization [78, 79]; (iv) confinement of T_1_-W Cas in molecular level to modulate their relaxation process [80, 81]. Li et al*.* synthesized Fe_3_O_4_/MnO hybrid nanoparticles through a step-by-step thermal decomposition method [82]. As wished, the T_1_-weighted imaging ability of MnO combined with the T_2_-weighted imaging ability of Fe_3_O_4_ were both inherited in this nanosystem, with an r_1_ value of 5.37 mM^−1^ s^−1^ and r_2_ value of 203.82 mM^−1^ s^−1^. Further conjugated with targeting peptide chlorotoxin (CTX), dual-modal MRI detection of glioma was achieved (Fig. 2e). In contrast, Liu et al*.* prepared Fe_0.6_Mn_0.4_O nanoflowers via a one-step thermal decomposition approach [83]. Apart from satisfying T_1_–T_2_ dual-modal imaging properties, the nanoflowers also showed enhanced saturation magnetization (Ms) and obvious magnetic hysteresis loop, compared to the counterparts, which indicated the great potential in magnetic hyperthermia therapy (MHT).

**Fig. 3:**
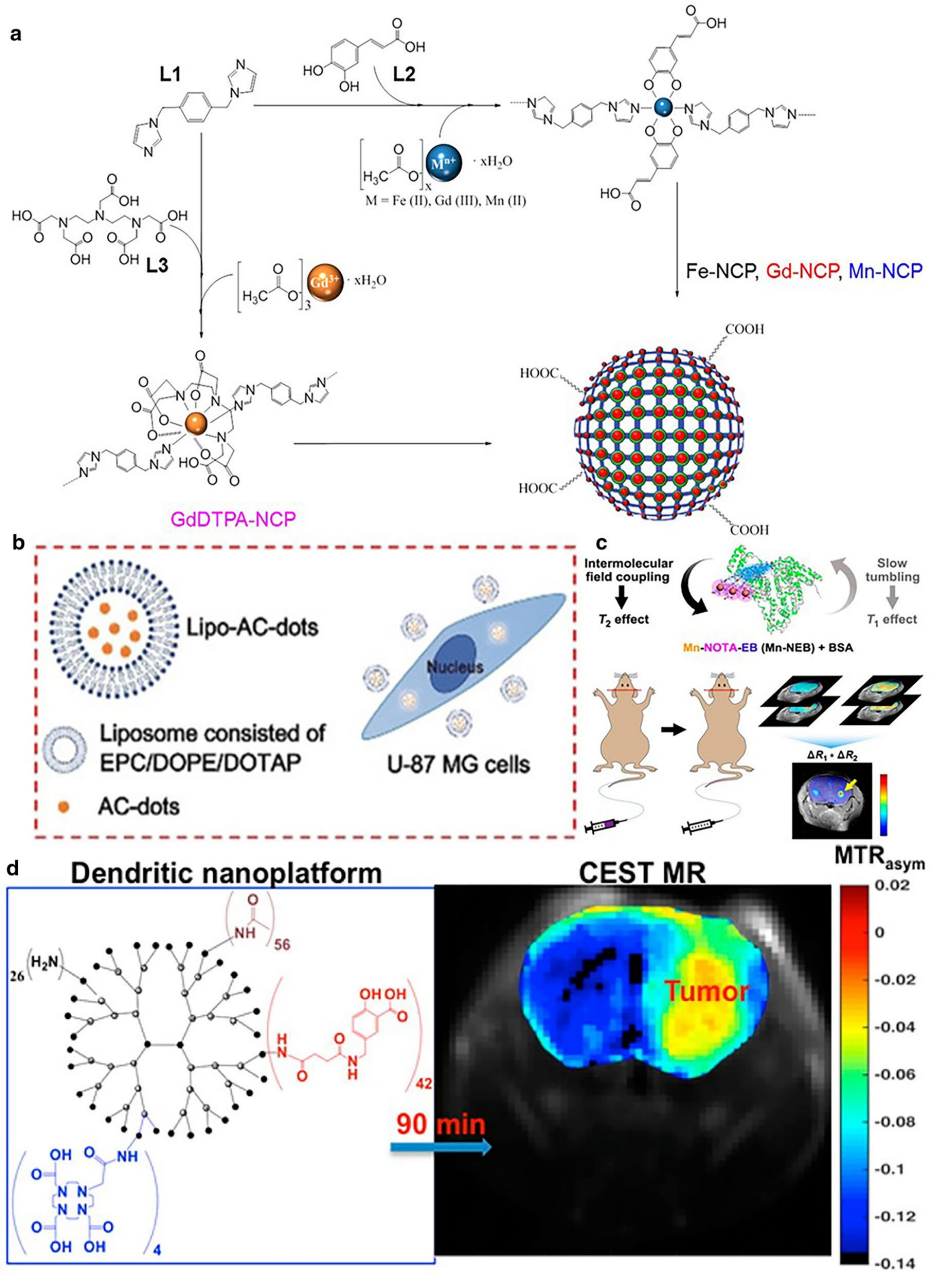
**a** Scheme for the synthesis of the different NCPs complexes. Reprint with permission [84]. Copyright 2018, American Chemical Society. **b** Schematic structure of the dendritic nanoplatform and its CEST MR performance. Reprint with permission [94]. **c** The intermolecular magnetic field coupling and slow tumbling feature of Mn-NEB + BSA lead to T1 and T2 relaxation enhancement. Reprint with permission [85]. Copyright 2019, American Chemical Society. Copyright 2016, American Chemical Society. **d** Schematic illustration of the cellular uptake of AC-dots following liposome encapsulation (Reprint with permission [95]. Copyright 2019, WILEY–VCH)

Owing to the inherent problems of inorganic nanomaterials, organic nanomaterials such as coordination polymers, are studied for serving as T_1_–T_2_ dual-modal Cas. A kind of ferrous ion-coordinated polymer has been reported to delineate glioma in both T_1_- and T_2_-weighted imaging mode [84], wherein one Fe^2+^ ion was coordinated with four oxygen atoms from catechol groups and two nitrogen atoms from imidazole groups, forming a six-coordination polymer. The catechol-based ligands benefited second-sphere interactions with water molecules that formed hydrogen bonding with the oxygen atoms of Fe–O–R linkages, which favored the enhancement of the T_1_ signal. Moreover, the metal ion center probably had coordination interactions with bound water molecules, which were not stable enough and exchanged with surrounding free water molecules, therefore influencing the relaxation time of water protons (Fig. 3a). Collectively, the Fe-coordinated polymer had an r_2_/r_1_ ratio of 2.1, which was eligible for proper dual-modal MRI agents for glioma detection. In another work, the researchers synthesized albumin-bound organic small molecules that chelated one Mn ion of each as the paramagnetic center [85]. The boosting of T_1_ relaxivity was achieved through the formation of macromolecules which impeded the tumbling of original small molecules. Simultaneously, the albumin-bound complexes modulated the intermolecular magnetic field coupling, which influenced the T_2_ relaxivity (Fig. 3b). Fulfilling the nanoprobe had an r_1_ value of 9.74 ± 0.55 mM^−1^ s^−1^ and r_2_ value of 71.8 ± 6.2 mM^−1^ s^−1^, appropriate for dual-modal MRI Cas. Besides, combined with quantitative computation of T_1_ and T_2_ relaxation time changes, a T_1_ and T_2_ mapping strategy was achieved, which was superior to conventional semi-quantitative signal-intensity-based MRI. As a result, not only the glioma region was effectively marked, but also the conventional false-positive region inside brain was potently eliminated.

### Nanomaterials for chemical exchange saturation transfer MRI

Chemical exchange saturation transfer (CEST) imaging is a newly developed MRI method based on chemical exchange theory and magnetization transfer (MT) technology. The CEST was originally found by Ward and his co-workers who were then researching MT in small molecules solution [86]. Different from traditional MRI contrast agents (Cas) which shorten the relaxation time (T_1_ or T_2_) of the surrounding water protons, Cas used for CEST MRI reduce the water signal through the chemical exchange of protons, which are pre-saturated by a radio-frequency pulse, from Cas to the ambient water molecules. By the echo-planar imaging (EPI) pulse sequence, the changes of water MR signal can be detected.

To generate obvious CEST effect, two conditions need to be met: (i) Δω ≥ K_ex_, in which Δω is the chemical shift of the protons in CEST Cas compared to that in water molecules and K_ex_ the chemical exchange rate of the protons; (ii) r_1_ ≤ K_ex_, where r_1_ stands for the longitudinal relaxation rate of the exchangeable protons. Concisely, condition (i) permits the desired resolution while condition (ii) allows the formation of enough pre-saturated protons in the Cas for exchange.

Generally, CEST Cas can be divided into endogenous and exogenous Cas according to the source, which can also be categorized as paramagnetic and diamagnetic Cas based on their magnetic properties. Due to the abundance of proton-contained functional groups (like –OH or –NH) in many endogenous molecules with proper Δω and K_ex_ (such as protein, polypeptide, and glucose), the CEST MRI can be conducted to determine the concentration of those molecules. Typically, the amide proton transfer (APT) phenomenon happens when protons in the amide group of proteins or peptides exchange with those of water molecules, which renders it possible to sensitively detect the slight concentration variations of those molecules in abnormal tissues. As a result, APT technology has become one of the most widely explored CEST modalities. To regulate the key parameters for CEST more precisely, exogenous Cas is also well studied, from diamagnetic molecules like sugar and amino acids to paramagnetic lanthanide agents and other kinds [87]. The presence of nanostructure shows great potential in modulating the exchange behavior of the pre-saturated protons, thus very promising for serving as nano Cas for CEST MRI, and in turn, the formed nano Cas become self-traceable for directly monitoring the in vivo behavior [88, 89]. Liposome, as one of the most well-known nanocarriers, is widely reported to enhance the performance of CEST Cas due to the existence of phospholipid bilayer, which affects the exchange process of the protons [89–92]. Han et al*.* synthesized a kind of injectable liposomal hydrogel with barbituric acid (BA) encapsulated inside for CEST MRI of the glioblastoma [93]. Interestingly, the liposomes and drugs exhibited distinct proton chemical shifts relative to water molecules (− 3.5 ppm for liposome and 5 ppm for BA drug), which implied the possibility of simultaneously monitoring the concentration changes of both at the tumor site. The release behavior of liposomes and drugs could be monitored over 3 days, realizing multiparametric imaging. Salicylic acid, a kind of diamagnetic molecules with many kinds of derivatives, was decorated onto the surface of generation 5-poly(amidoamine) (PAMAM) dendrimers to investigate the biodistribution after convection-enhanced delivery (CED) [94]. The nanoconjugates present the chemical shift of 9.4 ppm from the water with a tunable proton exchange rate, which was achieved by conjugation of different proton-involved functional groups. As expected, the nanoprobe could produce a strong CEST signal for over 1.5 h at the glioma site (Fig. 3c). In addition to organic nano Cas, hydrophilic carbon dots were also demonstrated to produce CEST signals due to the abundance of exchangeable protons on the surface [95]. The carbon dots were obtained through a microwave-assisted synthesis method with glucose as the carbon source and arginine as the hydrophilic modification molecules. Because of the presence of hydroxy groups and guanidine groups, an appreciable CEST MRI signal can be detected (1–2 ppm shift from water). By further liposome encapsulation, the carbon dots could differentiate the tumor region (Fig. 3d).

## Fluorescence imaging

Fluorescence imaging (FLI) is another extensively adopted imaging modality, which features high sensitivity, low cost, and real-time acquisition [96]. When fluorescent substances (such as some photosensitizers, for example, chlorin e6 and porphyrin) are excited by the external energy, the electrons in the ground state jump to a higher energy level and the whole system is in a metastable state, followed by electron transition back to the ground state in radiative transition (such as fluorescence and phosphorescence) or non-radiative transition (such as heat) pathways. A large variety of fluorescent probes have been developed for glioma imaging and detection.

### Organic fluorescent nanoprobes

**Fig. 4:**
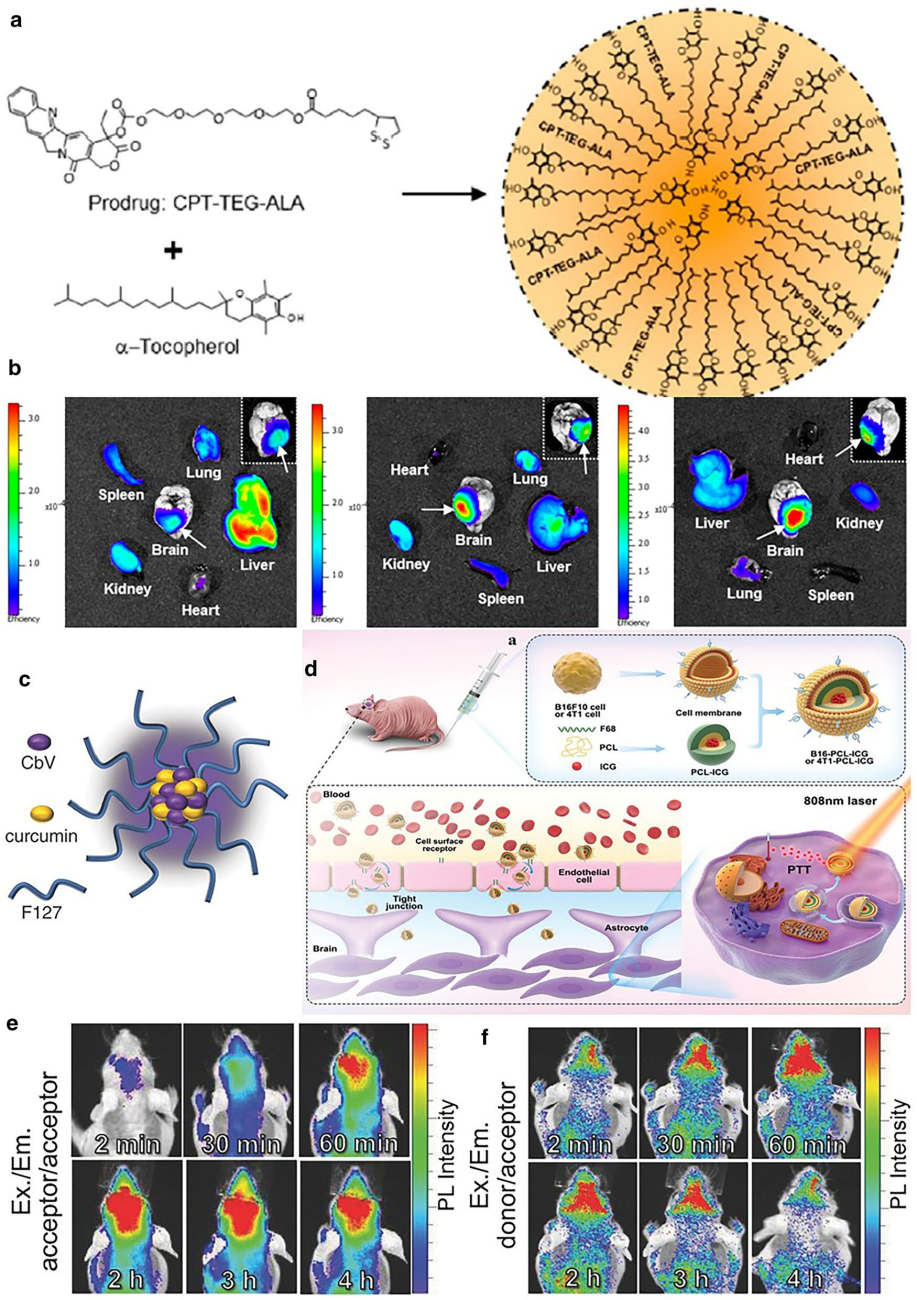
**a** CPT prodrug and R-tocopherol (Toco) undergo spontaneous emulsification into CPT-TEG-ALA/Toco nanoprodrug (CPT nanoprodrug). **b** Representative fluorescence images of brain and organs harvested from brain tumor-bearing mice (i) 5, (ii) 24, and (iii) 48 h after intravenous injection of Cy5.5-fluorescent CPT nanoprodrug. Reprint with permission [97]. American Chemical Society. **c** Schematic representation of TPN. **d** (i) The preparation process of biomimetic nanocarriers (B16-PCL-ICG or 4T1-PCL-ICG). (ii) Schematic illustration of the possible mechanism of biomimetic nanocarriers across BBB to brain tumors for imaging and photothermal therapy. Biomimetic nanocarriers bind cell surface receptors on the brain endothelial cells to mediate the transcytosis of nanoparticles across BBB. Reprint with permission [104]. Copyright 2020, Wiley–VCH. **e** In vivo imaging of temporal signals from TPN-cur/CbV intravenously injected in normal mice (n = 3). Excitation/emission wavelengths are 640 nm/700 nm (direct excitation) and **f** 500 nm/700 nm (energy-transferred indirect excitation). Imaging time points after sample injection are indicated (Reprint with permission [106]. Copyright 2016, Wiley–VCH)

Organic small molecule dyes are widely utilized in nanomaterials to serve as fluorophores, which are usually conjugated onto the surface of nanocarriers or encapsulated inside [97–102]. For example, Cy5.5, a kind of cyanine dye with Ex/Em of 675/695 nm, was reported to be decorated on the surface of camptothecin (CPT) nanoprodrug that could be activated by oxidative tumor microenvironment through the reaction between thiol group of the prodrug and maleimide group from the Cy5.5 derivative (Fig. 4a) [97]. The accumulation of the nanoprodrug in the glioma site was observed through the intravital imaging system (IVIS) (Fig. 4b). Also, Au nanoparticles conjugated on the surface of nano-sized gelatin were modified with Cy5.5 for glioma imaging [101]. Owing to the over-expression of matrix metalloproteinase-2 (MMP-2) in the tumor region, the gelatin can be biodegraded, leading to the shrinkage of nanoparticles, which further benefits the deep penetration. Cy5, another type of cyanine dye with Ex/Em slightly shorter (649/670 nm), was used to label polymer nanoparticles, poly (l-lysine)-grafted polyethyleneimine (PEI-PLL), by simple stirring for real-time monitoring of the distribution of the material [102]. Apart from cyanine dyes, DiR, a kind of lipophilic NIR fluorescent dye generally for cell membrane labeling, represents another frequently adopted probe (Ex/Em = 748/780 nm). Because of the prominent enhancement of the fluorescence intensity when combined with phospholipid or other lipophilic group-included macromolecules, DiR is usually conjugated onto polymer or protein nanoparticles [98–100, 103]. For instance, DiR was used to label poly (ethylene glycol)-poly (lactic acid) (PEG-PLA) nanoparticles modified with urokinase plasminogen activator (uPA)-sensitive cell-penetrating peptide (CPP) by hydrophobic interaction [103]. uPA is highly expressed in neo-vascular cells and glioma cells at the foci, closely associated with tumor growth, invasion, and metastasis. Hence, it is an ideal target for improving the accumulation of materials. As a result, the biodistribution and enrichment in the main organs and glioma site were distinctly visualized both ex vivo and in vivo*.*

In addition to acting as a tracer for locating the nanoparticles, organic fluorescent molecules also wield many other functions [104–109]. In a work reported by Kang et al*.*, a kind of mitochondria-targeted fluorescent probe was synthesized and confined in albumin molecules modified with folic acid (FA) to give desired nanoparticles [107]. Triphenyl phosphonium (TPP), a positively-charged molecule, was covalently conjugated with pheophorbide-a (PheoA), a porphyrin derivative that proved to be able to generate reactive oxygen species (ROS) under light irradiation, i.e. a kind of photosensitizer. Thus, FL imaging, organelle-targeting, and photodynamic therapy were integrated into one molecule. Moreover, some fluorescent probes can also be used as photothermal agents [110, 111]. For example, indocyanine green (ICG), an FDA-approved drug for examining liver function, is frequently exploited to be the fluorescent tracer as well as the photothermal molecule [112, 113]. As for glioma detection and treatment, the BBB traversing ability of nanoprobes is indispensable. One of the promising strategies that endow ICG with glioma targeting capacity lies in the coating of the cell membrane, especially from the cells that can migrate across BBB [104, 105]. Wang et al*.* selected murine melanoma cells (B16F10) and breast cancer cells (4T1) as the membrane source for ICG encapsulation, as both species may lead to brain metastasis. Normal cells (COS-7) were chosen as control. As expected, B16F10 and 4T1 cell membrane-enclosed ICG showed superior traversing efficiency compared to the COS-7 counterpart, contributing to efficient FL imaging of the glioma region and the subsequent photothermal ablation (Fig. 4c) [104].

However, the aggregation-caused quenching (ACQ) phenomenon is inevitable for common organic dyes, which greatly hinders their application. As a consequence, these fluorescent molecules are usually modified on the scaffold molecules of the nanoparticle to avoid severe aggregation, leading to insufficient loading amount. Thus, fluorescent molecules featuring solid-state fluorescence (SSF) or aggregation-enhanced fluorescence (AEF) are developed for biomedical imaging [114]. Classically, this kind of molecule has nonplanar geometries with torsional freedom, which favors the non-radiation transition of the electrons after light irradiation. When increasing the concentration of the molecules, the torsional motion is restricted, which benefits the enhancement of fluorescence. On this account, high loading capacity and quantum yield can be achieved at the same time. Besides, the generated fluorescence can be harnessed to precisely monitoring the drug release. In a work reported by Singh et al., CbV, a kind of SSF molecule based on the dipolar aryl vinyl (ArV) scaffold, was encapsulated inside F127 polymer with curcumin (Fig. 4d) [106]. Interestingly, the emitted fluorescence of curcumin could be well absorbed by surrounding CbV molecules, resulting in the fluorescence resonance energy transfer (FRET) effect. Accordingly, when the curcumin was unreleased, fluorescence at the wavelength of 700 nm was detected owing to FRET. Subsequently, the fluorescence gradually vanished along with the drug release (Fig. 4f), in contrast to the pharmacy fluorescence recovery (Fig. 4e). Collectively, the reported nanosystem not only accumulated efficiently in the glioma region but also well reflected the drug release for glioma treatment.

### Inorganic fluorescent nanoprobes

Inorganic fluorescent nanoprobes, such as quantum dots (QDs), represent another big category for biomedical imaging [115]. Compared to organic fluorescent molecules, inorganic nanoprobes feature better photostability against photobleaching and a high surface-area-to-volume ratio that permits the conjugation of functional molecules. Nevertheless, biodegradability, as well as metabolizability, is still a great challenge.

**Fig. 5:**
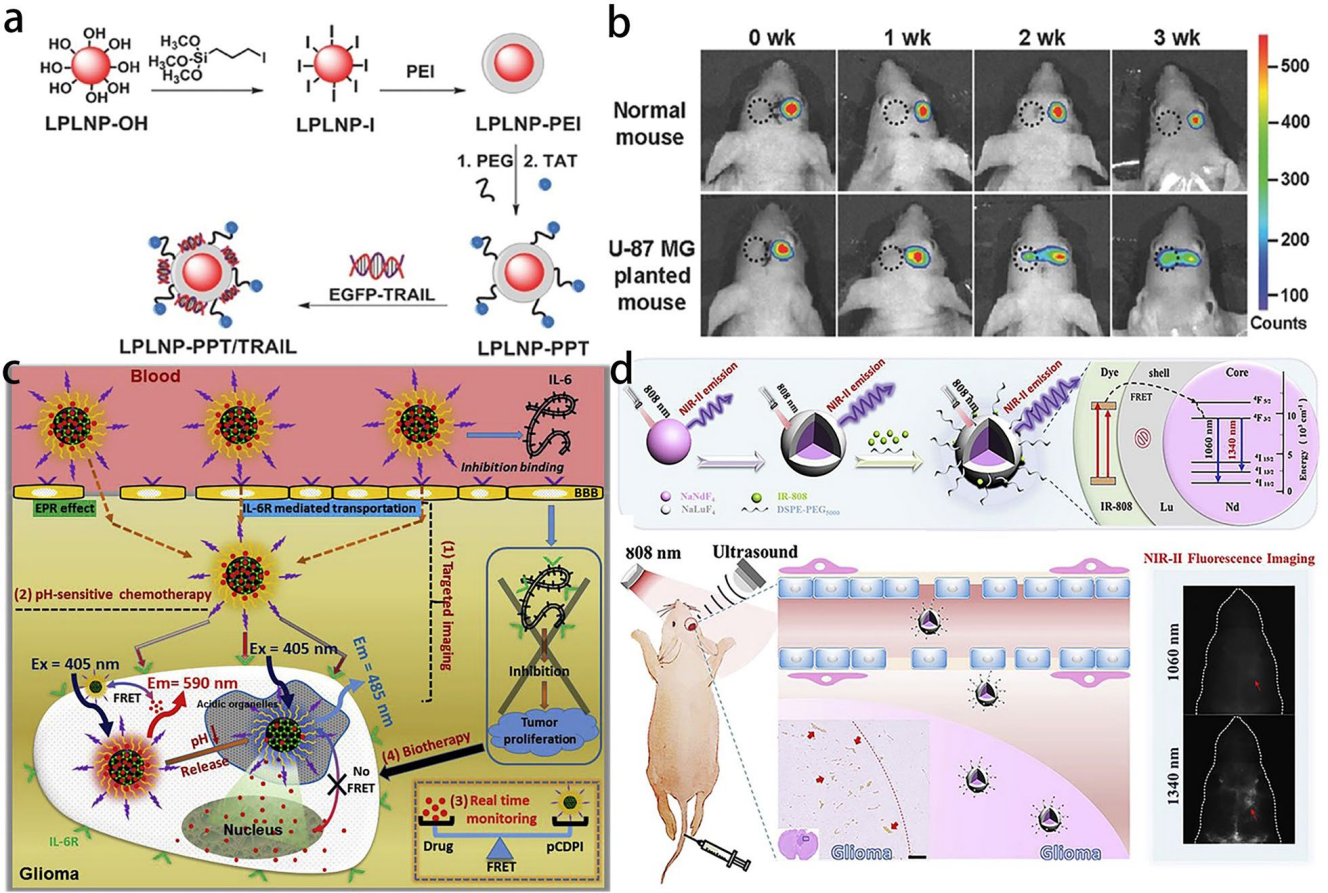
**a** Schematic representation of materials surface modification. **b** In vivo migration study of LPLNP-PPT labeled MSC. Reprint with permission [119]. Copyright 2017, Wiley–VCH. **c** Schematic diagrams of in vivo delivery process of multifunctional pCDPID and its application for targeted imaging, pH-sensitive chemotherapy, real-time monitoring, and biotherapy. Reprint with permission [124]. Copyright 2017, Elsevier. **d** (i) Schematic illustration of the synthesis of water-soluble dye-sensitized core–shell NaNdF_4_@NaLuF_4_/IR-808@DSPE-PEG5000 NPs and their energy transfer mechanism. (ii) Application of the core–shell NaNdF_4_@NaLuF_4_/IR-808@DSPE-PEG5000 NPs in NIR-II fluorescence imaging of orthotopic glioblastoma under the ultrasound-mediated opening of the BBB, and rare-earth staining of brain tissue after delivery into the brain (Reprint with permission [126]. Copyright 2019, Elsevier)

As for glioma imaging, these inorganic nanoprobes can be roughly divided into chalcogenide core/shell QDs, carbon QDs and other kinds of nanoparticles. Chalcogenide QDs, which are usually covered with a ZnS shell to improve the chemical and optical stability of the core materials, are widely studied [116]. CdSe/ZnS QDs with the maximum emission wavelength at 610 nm, were modified with aptamer for targeting [117]. Similarly, a kind of CdSeTe/ZnS QDs with a maximum emission wavelength at 800 nm, were combined with liposomes to achieve glioma imaging through a convection-enhanced delivery (CED) method [118]. However, the existence of a cadmium element severely hampered their further application as the Cd element is highly toxic to human bodies. Based on that, Liu et al*.* developed cadmium-free CuInSe_2_/ZnS QDs with Ex/Em of 650/709 nm, further modified with a targeting peptide CGKRK for accumulation in glioma site [115]. Moreover, it is worth mentioning that chalcogenide nanoparticles can also serve as long persistent luminescence nanoparticles (LPLNPs) for cell labeling. Different from transient fluorescence, LPLNPs can generate luminescence lasting for up to several hours after light irradiation, and the luminescence can be renewed by light-emitting diode (LED) light. Wu et al*.* developed a kind of LPLNP with the composition of Zn_1.1_Ga_1.8_Ge_0.1_O_4_:0.5%Cr^3+^, 0.5%Eu^3+^, which can produce luminescence at the wavelength of around 700 nm with a long time persistence [119]. Interestingly, after the red LED irradiation, the attenuated luminescence was well recovered. Thus, this kind of LPLNP conjugated with plasmid was utilized to label mesenchymal stem cells (MSCs) for gene transfection and the following glioma therapy (Fig. 5a). As expected, the LPLNP well monitored the migration of MSCs towards the glioma foci and achieved a satisfactory therapeutic outcome (Fig. 5b).

Carbon QDs represent another extensively studied category in inorganic nanoprobes for glioma imaging. Traditionally, the synthesized carbon QDs show excitation wavelength shorter than 500 nm and emission wavelength shorter than 600 nm, which is unsuitable for in vivo bioimaging [120]. Thus, developing carbon QDs with a longer excitation/emission wavelength has attracted a wide range of interest. Ruan et al*.* synthesized a kind of carbon QDs that could be excited under 560 nm with the emission wavelength of ~ 650 nm using glutamic acid and glucose as the precursor [121]. Angiopep-2 was anchored onto the surface for glioma targeting. Afterward, the synthetic route of carbon CDs was optimized by them, using glycine as the only precursor through a one-step heat treatment method [122]. The obtained carbon QDs also exhibited an emission wavelength longer than 600 nm with comparable quantum yield. Furthermore, a type of carbon QDs with self-targeting ability was fabricated using d-glucose and l-aspartic acid as the raw materials, followed by a straightforward pyrolysis process [123]. The final product displayed excitation-dependent full-color emission characteristics, similar to the above-mentioned kinds of carbon QDs, with an emission wavelength up to 635 nm (600 nm excitation). The innate targeting ability was speculated to be ascribed to the formation of RGD-like functional groups during synthesis. Besides, it was also reported that carbon QDs could be used to monitor the drug release through the FRET effect [124]. Carbon QDs were incorporated in a polymer nanoparticle with doxorubicin (DOX) molecules. The emission wavelength of carbon QDs at 485 nm could be efficiently absorbed by DOX, finally generating the fluorescence at the wavelength of 590 nm. When the DOX molecules were released from the nanoparticle, the FRET effect vanished. Thus, the drug release could be well monitored by detecting the fluorescence at 590 nm (Fig. 5c).

Apart from chalcogenide core/shell QDs and carbon QDs, there are also other kinds of inorganic fluorescent nanoprobes for glioma imaging [125, 126]. For example, Au nanoclusters (NCs) stabilized with zwitterionic molecules, were reported to conduct FL imaging of glioma [125]. The synthesized Au NCs exhibited an emission wavelength at around 800 nm, with the size of only ~ 2 nm, which could be expediently excreted through renal clearance as determined by advanced imaging techniques such as multi-elemental Laser-Induced Breakdown Spectroscopy (LIBS) imaging. Rare-earth nanoparticles, especially niobium (Nb)-contained species, have been demonstrated to exhibit both NIR-I and NIR-II fluorescence emission ability [127, 128], with the intensity in the NIR-II region much weaker. Liu et al. managed to solve this problem by two strategies: (i) surface coating of a NaLuF4 layer to reduce the surface effect and boost fluorescence emission, (ii) surface introduction of IR-808 organic molecules to enhance NIR light absorption for NIR-II fluorescence excitation. As expected, the evolved nanoparticles showed more than 10 times higher fluorescence intensity in 1340 nm than that of naked NaNdF_4_ nanoparticles, favoring deeper penetration and higher temporal and spatial resolution (Fig. 5d). Using focused ultrasound to temporarily open BBB, the nanoparticles efficiently accumulate in the glioma region, achieving the NIR-II fluorescence imaging.

### Fluorescent nanoprobes for intraoperative guidance

Although multiple novel therapeutic modalities have been developed, such as photothermal therapy (PTT), photodynamic therapy (PDT), and immunotherapy, surgical resection of the solid tumor is still considered in priority. However, due to the infiltrative nature of glioma, it’s hardly possible for surgeons to distinguish malignancies from normal tissues only by naked eyes. As a result, cancerous tissues can’t be removed completely, which is the main cause of recurrence. Therefore, developing real-time imaging techniques that can provide prompt and precise information about glioma is of great importance. Due to the high sensitivity and real-time imaging characteristics of FL imaging, it’s becoming a very promising imaging modality for intraoperative guidance. In recent years, quite a few fluorescent nanoprobes have been synthesized to provide intraoperative guidance for the complete elimination of glioma [129–133].

**Fig. 6:**
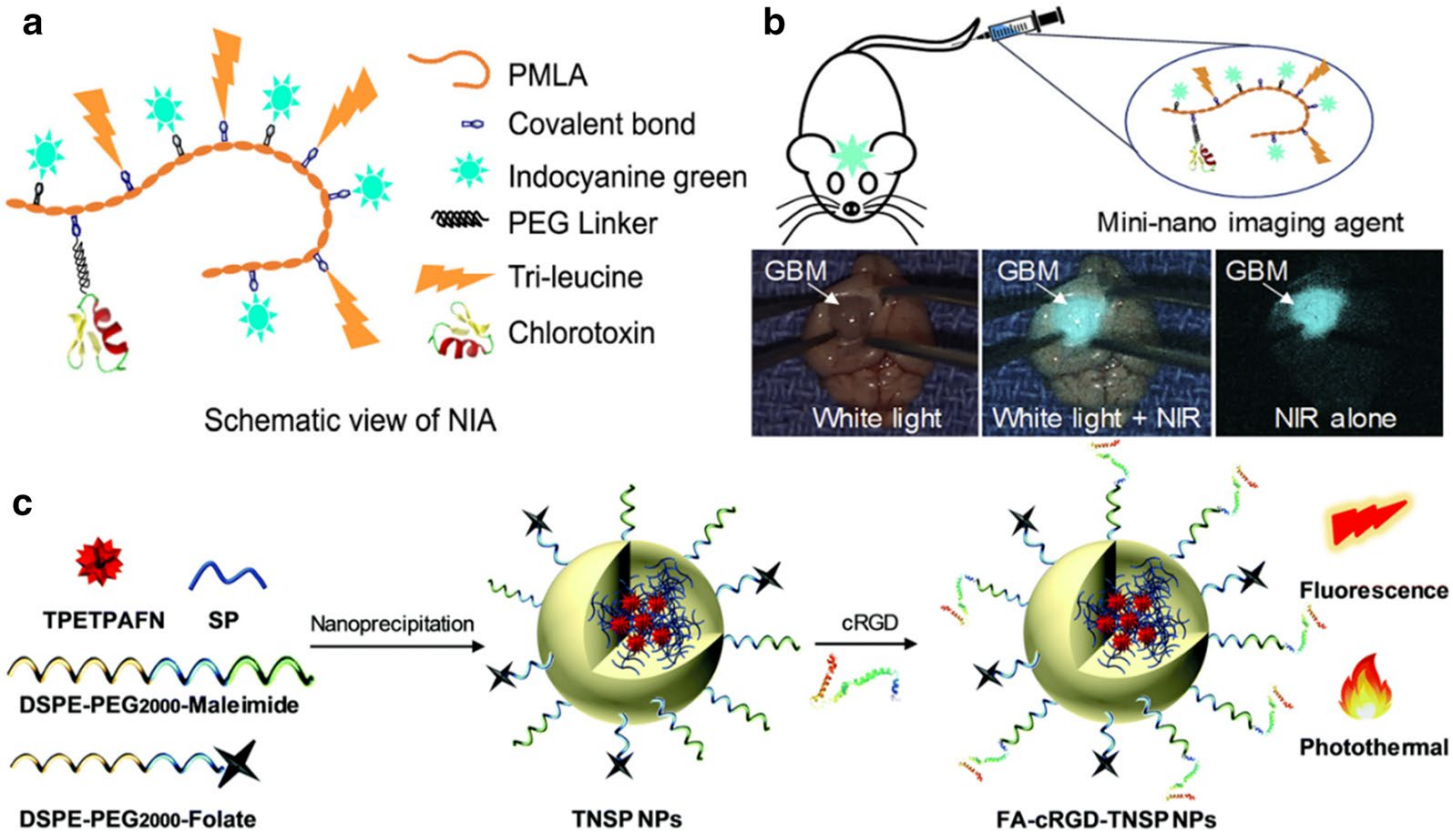
**a** Cartoon depicting the assumed functional organization of the imaging agent, represented by separation of fluorophores with hydrophobic side chains of the tri-leucine peptide. **b** The fluorescence imaging-guided resection of glioma under the assistance of SIRIS. Reprint with permission [132]. Copyright 2019, Elsevier. **c** Schematic illustration of the preparation process (Reprint with permission [133]. Copyright 2019, Royal Society of Chemistry)

Generally, to achieve this goal, the nanoprobes ought to have excellent targeting ability for glioma, which implies precise recognition of the tumor margin. Also, the high intensity of the excited fluorescence is indispensable for surgeons to distinctly locate the tumor. Patil et al*.* harnessed polymeric acid as the nanoplatform to conjugate functional molecules for intraoperative guidance [132]. Specifically, chlorotoxin was chosen as the targeting moiety while ICG acted as a fluorescent probe. Particularly, tri-leucine was introduced between ICG molecules to reduce the ICG aggregation-induced self-quenching and bring a hydrophobic microenvironment at the same time, which helped to enhance the fluorescence intensity (Fig. 6a). As expected, the nanocomposites potently surmounted BBB and were well accumulated in the glioma region with intense fluorescence signal, by which the resection process was finely guided, under the assistance of SIRIS (Synchronized near-InfraRed Imaging System) (Fig. 6b). Cai et al. developed a kind of FL nanoprobes that precisely delineate the tumor margin through a dual-targeting strategy [133]. Folate and cRGD peptide with the ratio of 1:3 modified on the surface was proved to be the optimal choice determined by confocal imaging results. TPETPAFN, a kind of typical aggregation-induced emission (AIE) molecule with a maximum emission peak at ~ 700 nm, was introduced to be fluorogenic (Fig. 6c). Besides, other kinds of organic fluorescent, such as Ce6 and DiD, are reported to wield the guidance function as well [130, 131]. In a work reported by Xu et al*.*, researchers found that Ce6 had a high affinity towards immunoglobulin G (IgG), which is much stronger than that towards human serum albumin (HSA), the endogenous carrier of Ce6. Based on that, a novel nanosensitizer termed Chloringlobulin comprised of Ce6, IgG and PVP was synthesized through a facile self-assembly approach. It had spherical morphology with a diameter of ~ 30 nm. Interestingly, the nanostructure enhanced the Ce6 accumulation in the tumor without changing its blood circulation half-life, which favored fluorescence-guided surgery combined with PDT. Besides, by further incorporating anti-PD-L1 antibodies during synthesis, immunotherapy was also achieved at the same time [130]. In another research, Guo and coworkers utilized engineered microglia for intraoperative guidance [131]. Specifically, microglial cells were firstly activated by citrate-stabilized iron oxide to inhibit the expression of M2 markers like CD206, which reduced the risks of immunosuppression. Simultaneously, up-regulated expression of apolipoprotein E, transferrin, and other ligands that benefit BBB crossing was also achieved. Furthermore, activated microglial cells were loaded with near-infrared fluorescent dye DiD for fluorescence-guided surgery. Results showed that the engineered microglia produced an intense fluorescence signal in the tumor region for a prolonged period (4–24 h), contributing to elaborate operations. As for inorganic nanoprobes, however, much fewer inorganic nanoprobes were involved in this field nowadays, probably because of the biosafety-related issues.

## Photoacoustic imaging

Photoacoustic imaging (PA), which takes the advantage of light (usually in the NIR region for deeper penetration) as the excitation source and the induced acoustic waves as the emission source, has emerged as a novel imaging modality in the biomedical field [134–138]. PAI combines the merits of optical imaging (high sensitivity and selectivity) and ultrasound imaging (deep penetration). Furthermore, it avoids light scattering that is inevitable in optical imaging, which would compromise the spatial-resolution. Therefore, PAI is becoming a very promising imaging method. Cas used for PAI require strong light absorption and turn the light energy into heat, which is similar to the photothermal agents for PTT in principle. Besides, heat-induced expansion is another vital factor, which directly triggers the generation of ultrasound waves. Accordingly, nanomaterials with intense NIR light absorption, high photothermal conversion coefficient, and appropriate elastic properties are the ideal Cas for PAI.

In general, PAI Cas can be divided into organic Cas, like small molecule-based nanoprobes and conjugated polymers, and inorganic Cas, like MoS_2_ nanosheets and Cu-based nanoparticles. Quite a few PAI Cas also possess other imaging abilities such as MRI or CT, which will be introduced in multimodal imaging nanoprobes-related chapters.

**Fig. 7:**
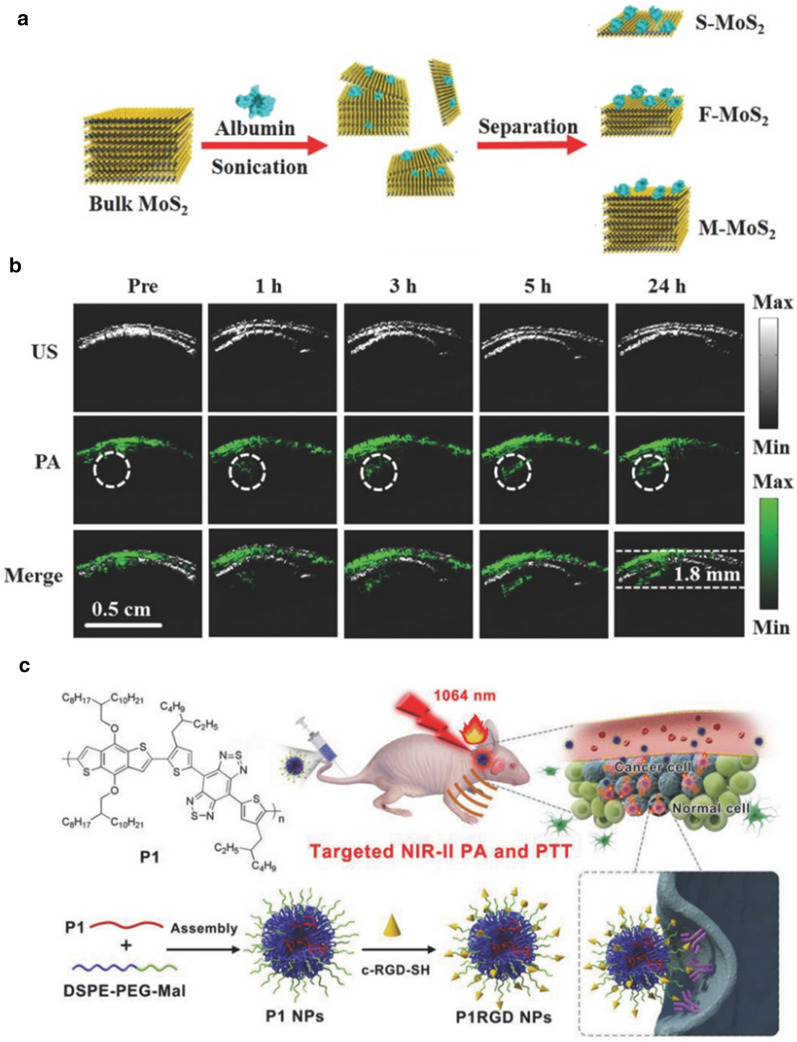
**a** Schematic illustration of the synthesis procedure of MoS_2_ nanosheets with various layered nanostructures. **b** B-scan PA images of the brain tumor region obtained before (Pre) and 1, 3, 5, and 24 h after i.v. injection of S-MoS_2_. US images delineated the skin and tumor boundaries, while the PA images showed the accumulation and distribution of S-MoS_2_ within the tumor region. Reprint with permission [139]. Copyright 2016, Wiley–VCH. **c** Illustration of nanoparticle fabrication and in vivo brain tumor photothermal therapy and photoacoustic imaging (Reprint with permission [148]. Copyright 2018, Wiley–VCH)

Wang et al*.* systematically investigated the influence of layer numbers on the PA performance of MoS_2_ nanosheets [139]. Three kinds of MoS_2_ nanosheets, which are single-layered (S-MoS_2_), few-layered (F-MoS_2_), and multiple-layered (M-MoS_2_), were synthesized through an albumin-assisted exfoliation method (Fig. 7a). Interestingly, along with the decrease of the layer number, the NIR light absorption was enhanced drastically, with the maximum PA signal achieved in S-MoS_2_. The improved adsorption could be ascribed to the direct excitonic transitions at the K point of the Brillouin zone, according to the relevant research [140]. Besides, the reduction of the layer number contributed to the improvement of elasticity, which favored mechanical vibration after light absorption. Collectively, the S-MoS_2_ managed to produce an obvious PA signal at ~ 1.5 mm below the skull under 808 nm laser irradiation (Fig. 7b). Zhang et al*.* reported a kind of Cu_2−x_Se nanoparticles with strong light absorption in the NIR-II region (1064 nm) for PAI-guided PDT [141]. Focused ultrasound was applied to temporarily open BBB for the enrichment of Cu_2−x_Se in the glioma site. Moreover, Prussian blue nanoparticles, gold nanostars, and many other inorganic nanomaterials are reported to act as PAI Cas for glioma imaging [142, 143].

Conjugated polymers, which comprise electron acceptors (A) and electron donors (D), have attracted extensive interest due to the distinctive electron properties and hold great promise in biomedical applications [144–146]. Guo et al*.* explored the effects of D–A strength on the light absorption, emission, and extinction coefficient that are vital for PA performance [147]. It was found that stronger D–A interactions favored intramolecular charge transfer (ICT), red-shifted absorption, and photothermal conversion, thus beneficial for PA imaging. Density functional theory (DFT) and the time-dependent DFT (TDDFT) calculations were further implemented to explain the underlying mechanisms. Results showed that the planar backbone structure of the conjugated polymers facilitated Intra/interchain electronic interactions, which benefited long-wavelength absorption. Besides, the oscillator strength improved with the increase of D–A interaction, which led to a high extinction coefficient. Based on the theoretical analysis, the research group further synthesized a kind of conjugated polymer with a strong absorption band in the NIR-II region. cRGD was modified on the surface for glioma targeting (Fig. 7c) [148]. As expected, the nanoprobes could locate the tumor at a depth of ~ 3 mm below the skull with an ultra-high signal to background ratio (SBR) of 90 (1064 nm laser), proving the great potential as the PAI CA. In addition to conjugated polymers, J-aggregates of cocaine dyes [149], dendrimers [150], and other kinds of polymers [151, 152] are also actively involved in this field.

## Nuclear medical imaging

Nuclear medical imaging, which refers to Positron Emission Tomography (PET) and Single-Photon Emission Computed Tomography (SPECT), accounts for a crucial part in the molecular imaging field. For PET imaging, positron-emitting radioactive isotopes (such as ^18^F, ^11^C, or ^15^O) labeled pharmaceuticals (termed radio-pharmaceuticals) are administered to the patient, and with the decay of the isotope, positrons are emitted. When one positron encounters one electron, the annihilation process happens with the generation of two gamma photons in the opposite direction, through which the information of the foci is well-reflected. As to SPECT imaging, the whole process is similar. Gamma photon-emitting radioactive isotopes (such as ^99m^Tc, ^201^Tl, or ^123^I) labeled pharmaceuticals are utilized and during the decay process, every radio-molecule emits one gamma photon that is detected to be the indicator of the state of illness.

Nuclear medical imaging reflects the pathological changes at the molecular level before deteriorating into clearly evident structural alterations of the organs and tissues. Also, the outcomes of the therapy can be sensitively monitored, benefiting the following possible optimization of the therapeutic regimen. Classically, PET features higher sensitivity and resolution as well as the feasibility for precise quantitative analysis while SPECT is more cost-effective and has greater applicability due to the existence of more radioisotopes with suitable half-life.

For PET imaging, 2-deoxy-[^18^F]fluoro-d-glucose (^18^FDG), a kind of ^18^F labeled glucose analog, is widely used in the clinic for the detection of a brain tumor in which the glucose transporters and glycolytic enzymes are over-expressed compared to that in normal tissues [153]. Apart from this, ^11^C-Methyl-methionine (MET), ^11^C, or ^18^F. labeled choline and ^18^F-labeled aromatic amino acid analogs are also reported to serve as tumor-specific radio-pharmaceuticals [153–155]. ^18^F has a longer half-life (110 min) than other common radioisotopes like ^11^C (15 min), ^13^N (10 min), or ^15^O (2 min), therefore not requiring the on-site cyclotron for synthesis and being commercially available [156]. Additionally, metallic radioisotopes such as ^64^Cu, ^68^Ga, ^86^Y, and ^89^Zr represent another category in PET imaging. Compared to the nonmetallic counterparts, a longer half-life (68 min for ^68^Ga and 12.7 h for ^64^Cu, for example) benefits the transportation of the produced radio-tracers and further application. Besides, metal elements can be easily chelated by various kinds of ligands, thus favouring the effective labelling. For example, Peng et al. synthesized a kind of fullerene-based nanoplatform with cyclic RGD (cRGD) decoration for glioma targeting [157]. Specifically, 1,4,7-triazacyclononane-1,4,7-triacetin acid (NOTA) was conjugated on the surface of the fullerene scaffold, serving as the ligand for ^64^Cu chelating. This nanoplatform managed to be accumulated in the glioma site and could be readily eliminated through the kidney. Apart from chelating, ^64^Cu can also be directly integrated into the nanomaterials for PET imaging [158, 159]. L-type amino acid transporters, which transport branched and aromatic amino acids, are highly expressed on several cancer types including glioma. Nodwell et al. developed a facile strategy for direct ^18^F‑fluorination of the unactivated C–H bonds of branched and aromatic amino acids through a decorating state-catalyzed process, which provides a feasible method for obtaining glioma-targeting peptides in PET imaging [160].

**Fig. 8:**
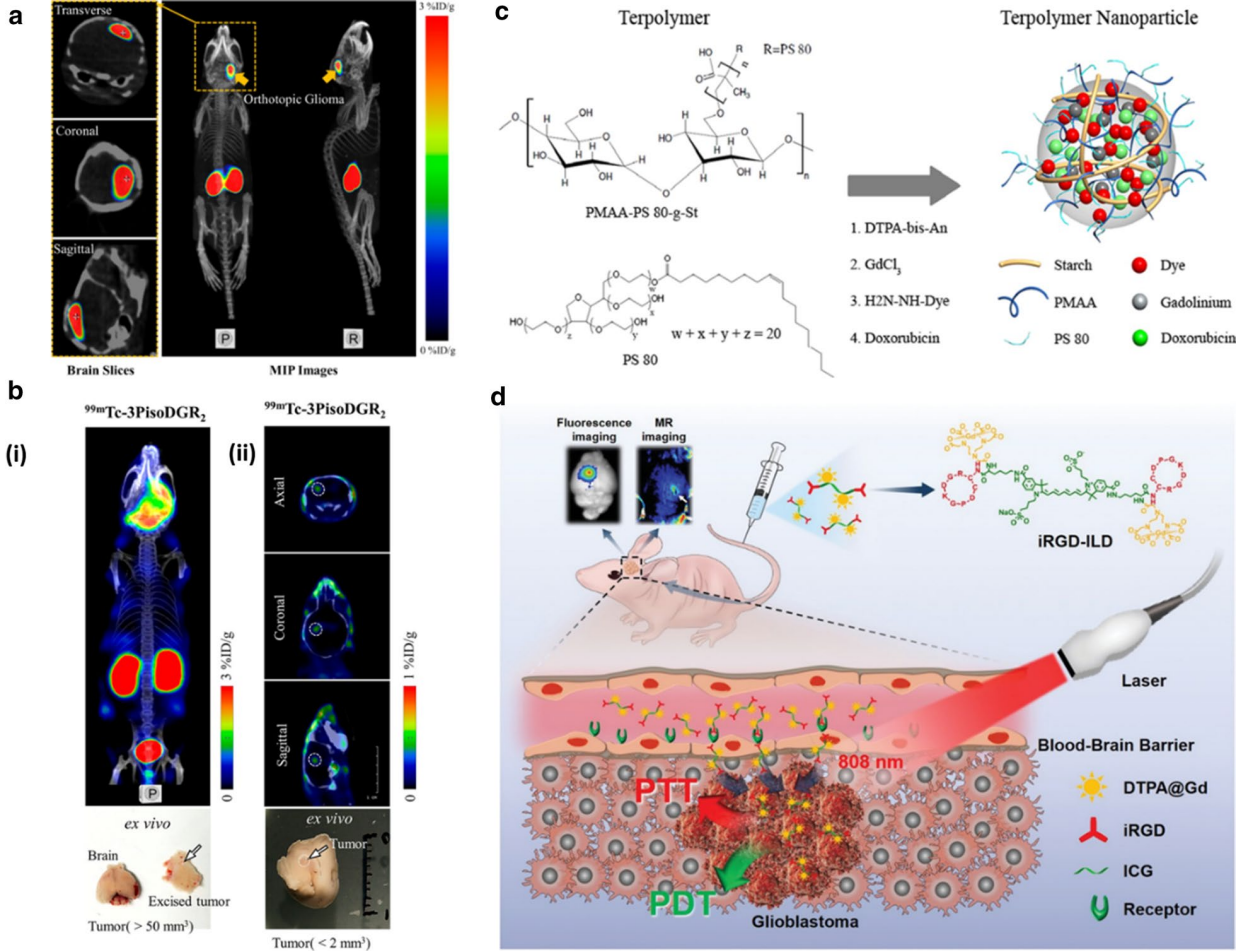
**a** Representative small-animal Nano SPECT/CT MIP images and brain slice images (including transverse, coronal, and sagittal sections) of 99mTc-HisoDGR in an orthotopic U87MG glioma tumor model obtained at 0.5 h p.i. Arrows and crosshairs indicate tumor location. For all the SPECT images, the bladder was excluded during imaging reconstruction. (P represents a posterior view; R represents the right lateral view.) Reprint with permission [163]. Copyright 2016, American Chemical Society. **b** (i) Representative maximum intensity projection image of 99mTc-3PisoDGR2 at 1 h p.i. with the tumor size > 50 mm^3^. (ii) Representative sectional images of 99mTc-3PisoDGR2 at 1 h p.i. with the tumor size < 2 mm^3^. Reprint with permission [161]. Copyright 2019, American Chemical Society. **c** Structures of the terpolymer and PS 80, and a schematic diagram of the self-assembly of PMAAPS 80-g-St terpolymer into nanoparticles upon conjugation of the fluorescent moieties, chelation with Gd^3+^, and complexation with doxorubicin. Reprint with permission [168]. Copyright 2014, American Chemical Society. **d** Schematic illustration of the molecular probe (iRGD-ILD) crossing BBB via α_v_β_3_ integrin-receptor-mediated transcytosis to enable bimodal MR/fluorescence imaging-guided photothermal/photodynamic therapy of intracranial glioblastoma (Reprint with permission [170]. Copyright 2020, Wiley–VCH)

As to SPECT imaging, gamma-emitting small molecules like ^99m^Tc-sestamibi (^99m^Tc-MIBI) and ^123^I-iodine-α-methyl tyrosine (^123^IMT) are commonly adopted. Wang’s group developed a series of ^99m^Tc-labeled targeting peptide-based nanoprobes for glioma SPECT imaging [161–163]. cRGD peptide, which has a high affinity towards integrin α_V_β_3_ that is over-expressed on glioma cells and endothelial cells of the tumor neovasculature, was modified and exploited for SPECT imaging of glioma [162]. Dimers of cRGD with improved uptake characteristics were synthesized with a further modification of ligands like isonicotinic acid, trisodium triphenylphosphine-3,3′,3″-trisulfonate (TPPTS), which functioned as the modulator of biodistribution. Different kinds of co-ligands were chosen and compared to systematically study the in vivo behaviors of the nanoprobes. As a result, the TPPTS-conjugated nanoprobe had the best property for glioma imaging. In addition to α_V_β_3_, there are also other kinds of integrins correlated well with glioma proliferation and invasion, among which α_5_β_1_ integrin is associated with high-grade glioma with poor prognosis. As a mimic of Arg-Gly-Asp (RGD) peptide, is RGD derivatives can recognize different kinds of integrins under varied conformation and molecular scaffold. Based on this, a kind of nanoprobe mainly comprising cyclic HEPA-peptide c (png-RGD) with flanking residues was developed to selectively target α_5_β_1_ for SPECT imaging of glioma [163]. As expected, the tumor region was highlighted at 0.5 h after intravenous (i.v.) injection and lasted for more than 2 h (Fig. 8a). To further improve the tumor-targeting ability as well as pharmacokinetics properties, dimerization, and albumin-binding strategies were adopted to modify the above-mentioned α_5_β_1_-targeting nanoprobe [161]. Consequently, the amount of tumor uptake was increased 2 times with prolonged blood circulation time. Moreover, tiny gliomas (< 2 mm^3^) could also be efficiently detected, thus very the potential for further clinical applications (Fig. 8b).

## Nanomaterials for multimodal imaging of glioma

Although tremendous progress has been made, from theory to technology, to improve the performance of each kind of imaging modality, it’s still far from meeting the ever-increasing demand for obtaining precise pathological information at the cellular or molecular level. MRI is one of the most powerful imaging methods with a superb spatial resolution (~ μm) and no penetration limits, which can well reflect the anatomical changes in abnormal tissues, especially water-rich soft tissues like the brain. However, the relatively low sensitivity (μM–mM) requires the administration of contrast agents with a high dosage, which may cause severe bio-safety issues. Optical imaging modalities like FL and chemiluminescence (CL) feature excellent sensitivity (pM–nM) but poor spatial resolution and limited tissue penetration, particularly unfavorable for the brain with skull protection. Despite the newly developing FL imaging technology in the NIR-IIb range (1500–1700 nm) with penetration depth up to several mm and spatial resolution of several μm, it’s still in its infancy with many unresolved problems [164–166]. Similarly, PA imaging, nuclear medical imaging as well as X-ray CT imaging also suffer from unsatisfying spatial resolution (usually at ~ mm) and other shortcomings. Therefore, integrating multiple imaging modalities into one system can take advantage of each imaging component, representing a feasible strategy to realize a precise diagnosis. The advancement of nanotechnology provides a solution to developing multimodal imaging agents. The tunable structure, abundant functional groups, and large surface-to-volume ratio (S/V) of the nanocarrier make it convenient to combine different imaging agents without mutual disruption. Moreover, the nanoparticle itself could also serve as various contrast agents through rational design. In this chapter, the progress of nanomaterials for multimodal imaging of glioma in recent years is summarized.

### Nanomaterials for dual-modal imaging of glioma

#### Nanomaterials for MR/FL imaging

As mentioned above, MRI is characterized by high spatial resolution and unlimited penetration depth while FL imaging is a very sensitive imaging modality. Therefore, MR and FL imaging are well complementary, and nanomaterials integrating them are the most explored nano Cas in multimodal imaging.

Generally, nanoprobes for MR/FL dual-modal imaging can be categorized as organic, partially organic, and inorganic, according to the imaging constituent. For organic nanoprobes, MR imaging is often achieved by manganese or gadolinium complexes such as Gd-DTPA, and FL imaging is realized through the introduction of organic fluorophores like Cy5.5. Li’s group synthesized a dendrimer-based nanoplatform for glioma imaging wherein Gd-DOTA acted as MRI CA and Cy5.5 as FLI CA [167]. Through the surface modification of cRGD and Angiopep-2 which targets integrin α_V_β_3_ and low-density lipoprotein receptor-related protein (LRP) receptors respectively, the nanoprobe could successfully traverse the blood–brain barrier and accumulate in the glioma region, precisely delineating the tumor boundary. Li et al*.* adopted poly(methacrylic acid)-polysorbate 80-grafted-starch as the scaffold of the nanocarrier to encapsulate Gd ions, doxorubicin, and a kind of NIR dye HiLyteFluor 750 for the imaging and treatment of brain metastases of breast cancer [168] (Fig. 8c). Polysorbate 80 was reported to be able to adsorb apolipoprotein-E (ApoE) in the plasma, which targets low-density lipoprotein receptors. Both T_1_-weighted MR imaging and NIR FL imaging indicated that the nanoparticles with the size of ~ 60 nm could well locate the tumor sites inside the brain at 30 min following intravenous injection. The immunofluorescent staining results showed that the released DOX induced severe apoptosis of the glioma cells. In addition to synthetic polymers, natural biomacromolecules like albumin were also reported as nanocarriers to chelate Mn^2+^ and combine sonosensitizers for focused ultrasound-induced thermal and sonodynamic therapy [169]. Moreover, Shuai’s group reported a kind of carrier-free nanoprobe, wherein indocyanine green (ICG) was conjugated with cRGD and Gd-DTPA to conduct MR/FL imaging-guided phototherapy (Fig. 8d) [170]. A two-photon confocal laser scanning microscope (CLSM) was used to directly observe the BBB penetration process of the nanoprobe. As anticipated, the clear extravasation of the nanoprobe from the vasculature in brain parenchyma was detected through red fluorescence at 10 min post-injection, with the intensity gradually increasing in the 1 h observation stretch. In contrast, the nanoprobe without RGD modification showed poor performance in BBB crossing, which is consistent with the MR imaging outcomes. Furthermore, efficient accumulation in the glioma site was confirmed by CLSM and intravital imaging system. Under 808 nm laser irradiation, the growth of orthotopic glioma was potently suppressed.

**Fig. 9:**
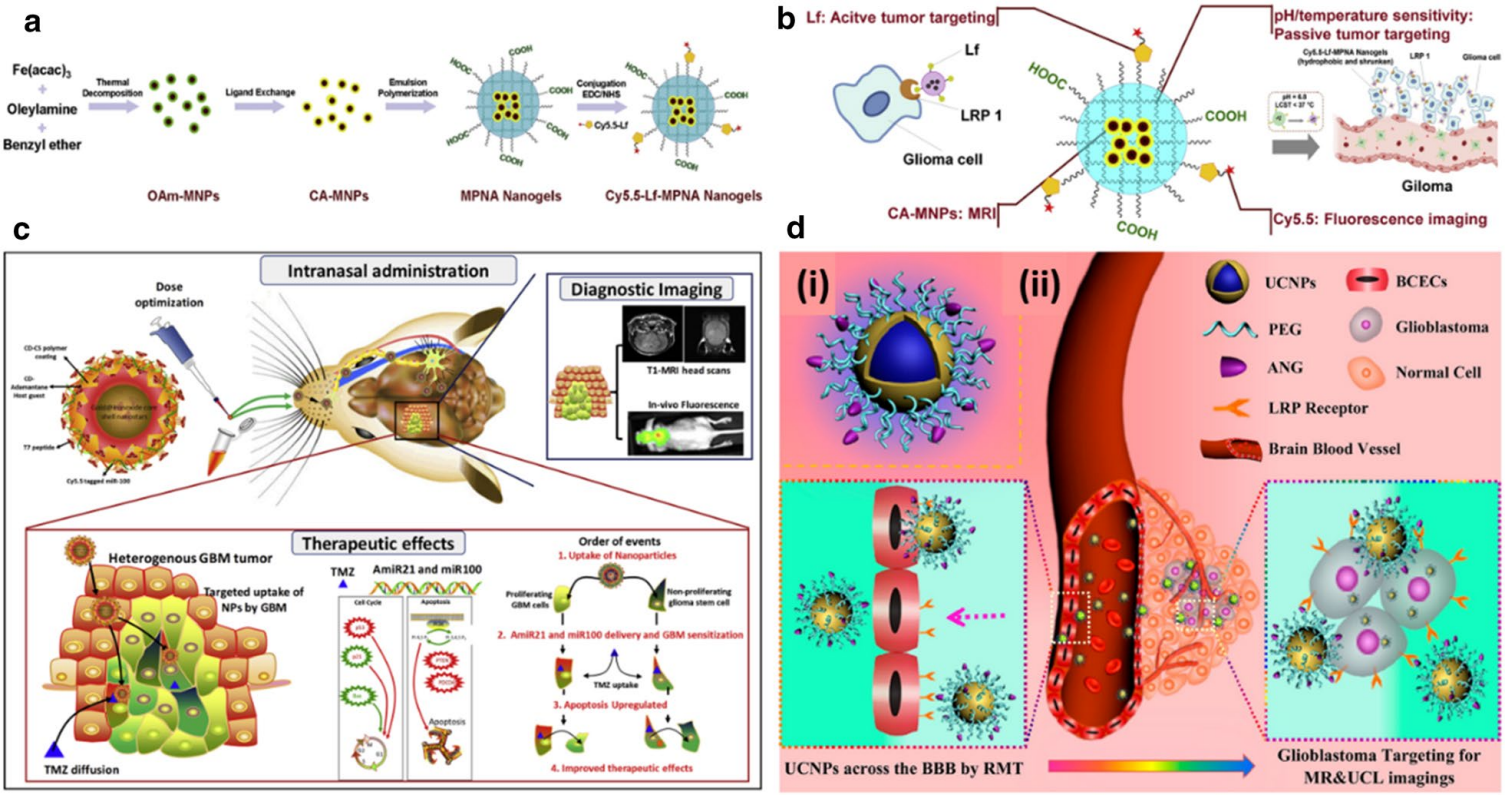
**a** Steps for preparation of Cy5.5-Lf-MPNA nanogels. **b** Illustration of the functioning mechanisms of Cy5.5-Lf-MPNA nanogels. Reprint with permission [172]. Copyright 2014, Elsevier. **c** Schematic illustration of the principle of intranasal polyGION-miRNAs delivery and its theranostic imaging and therapeutic effect in GBM in vivo. Reprint with permission [171]. Copyright 2019, Elsevier. **d** (i) Design of the dual-targeting ANG/PEG-UCNPs. (ii) Schematic diagram of the ANG/PEG-UCNPs as the dual targeting system to cross the BBB and target the glioblastoma via LRP mediated endocytosis, enabling MR and UCL imaging of intracranial glioblastoma (Reprint with permission [177]. Copyright 2014, American Chemical Society)

As to organic–inorganic hybrid nanoprobes, superparamagnetic iron oxide (SPIO) nanoparticles are usually used as the core to achieve T_2_-weighted MRI and organic fluorescent dyes as the FLI agents [171–175]. Jiang et al*.* reported a kind of polyacrylamide-based nanoplatform in which citric acid-coated SPIO nanoparticles were encapsulated inside [172]. Cy5.5-labeled lactoferrin was further conjugated on the surface to be the targeting moiety and FLI agents (Fig. 9a). Interestingly, the nanoplatform exhibited pH/temperature dual-responsive property. In physiological conditions, the nanoplatform was hydrophilic and swollen while in the tumor region, the hydrophobicity increased with the size becoming smaller, which could be ascribed to the decreased ionization degree of the carboxyl groups in weakly acidic conditions. The rising temperature could induce a similar variation. As a result, this property favored prolonged blood circulation time and enhanced enrichment in the foci (Fig. 9b). At 48 h post-injection, the nanoprobe produced striking signal contrast in the tumor with normal brain tissues, according to the MR and FL imaging results. The composition of the nanocarrier scaffold can also be another sort of polymers, such as chitosan, polyethylene glycol (PEG)-b-polycaprolactone (PCL) copolymer, liposome [173–175]. Sukumar et al*.* fabricated a type of multifunctional nanoformulation for intranasal delivery to deal with glioblastoma [171]. In detail, the gold-iron oxide core–shell nanostars were used as the core, with the surface decoration of β-cyclodextrin conjugated chitosan. Afterward, two kinds of miRNA, antimiR-21 and miR-100, were loaded onto the shell to suppress the expression of miR-21 and polo-like kinase 1 (PLK1), respectively, which could induce the apoptosis of p53 defective cells including glioblastoma cells. Moreover, the loaded miRNA could also potentiate the effects of temozolomide (TZM) for combination therapy. Finally, PEGylated T7 peptide was modified on the surface through CD-adamantane host–guest chemistry for targeting delivery. Cy5 dye was complexed with miRNA for FL imaging (Fig. 9c). Following intranasal administration, the tumor region was well identified by MR and FL signals. The coloaded miRNA successfully intervened in the apoptosis-relevant signaling pathways, as proved by immunoblot analysis. Consequently, the nanoformulation combined with the TMZ regimen reached the best results under the monitoring of MR and FL imaging. Apart from organic dyes for FLI, quantum dots are also involved in this hybrid nanosystem. For example, Li et al*.* synthesized an Ag_2_S-based nanoprobe in which Ag_2_S quantum dots as the core were conjugated with Gd-DOTA on the surface for preoperative diagnosis and intraoperative visualization [176]. This nanoprobe smaller than 10 nm in diameter exhibited strong fluorescence emission at the wavelength of 1200 nm and an adequate longitudinal relaxation rate of 4.9 mM^−1^ s^−1^, endowing it with MR/FL imaging capacity. 3T MRI results showed that the signal intensity reached a maximum at 10 h post-injection and the bright contrast signal could last for more than 2 days. Undergoing craniotomy, the tumor region was easily distinguished from the surrounding normal brain under NIR-II FL imaging, which provided precise guidance for surgical resection.

Inorganic nanoparticles are also actively involved in this application. Ni et al*.* synthesized a type of core–shell upconversion nanoparticle (UCNP) wherein NaYF_4_:Yb/Tm/Gd constituted the core and NaGdF_4_ the shell [177]. UCNP was further modified with PEG to improve the colloidal stability and angiopep-2 for glioma targeting. The introduction of the Gd element endowed the UCNP with MRI potential and the intrinsic characteristic of upconversion luminescence in the NIR region (excitation: 980 nm; emission: 800 nm) allowed FL imaging (Fig. 9d). After intravenous injection, the T_1_-weighted MR signal of the brain was detected at certain time points. Results indicated that the UCNP nanoprobe was accumulated the most at 1 h post-injection, with the signal gradually decreasing afterward. Correspondingly, the FL intensity also reached a maximum at the same time point, well locating the tumor according to the ex vivo fluorescent images. Besides UCNP, other inorganic nanoprobes such as Mn-doped carbon dots were also reported as MR/FL dual-modal imaging agents.

#### Nanomaterials for MR/PET (or SPECT) imaging

As mentioned before, MRI provides anatomical information of the abnormal tissues while nuclear medical imaging shows the metabolic changes at the molecular level. Thus, combining MRI with PET or SPECT imaging could obtain structural and functional information at the same time. Moreover, MRI features satisfying resolution while PET or SPECT has excellent sensitivity, which renders the combination very meaningful. Recently, the hybrid system that integrates MRI and PET devices has been used in the clinic for precise diagnosis. Due to the great potential of nanomaterials in biomedical applications, efforts have been paid to explore nanoprobes for MR/PET or MR/SPECT imaging of glioma [178–181]. For example, Chen et al*.* synthesized a gadofullerene-based nanoplatform for MR/PET imaging of glioblastoma [181]. Gd@C_82_ was labeled with ^64^Cu or ^89^Zr through the chelation of different ligands (like DOTA for capturing ^64^Cu) for PET imaging. cRGD was further modified on the surface for targeting. At 24 h post-injection, both MR and PET imaging revealed that the nanoprobe was efficiently accumulated in the tumor region, demonstrating its capacity for dual-modal imaging. Furthermore, the metabolic process of the nanoprobe was also investigated. By using ^89^Zr labeled nanoprobe, the biodistribution can be monitored for up to 30 days. Currently, the related research mainly focused on the subcutaneous model, with more efforts needed to explore nanoprobes for orthotopic glioma.

#### Nanomaterials for MR/CT imaging

**Fig. 10:**
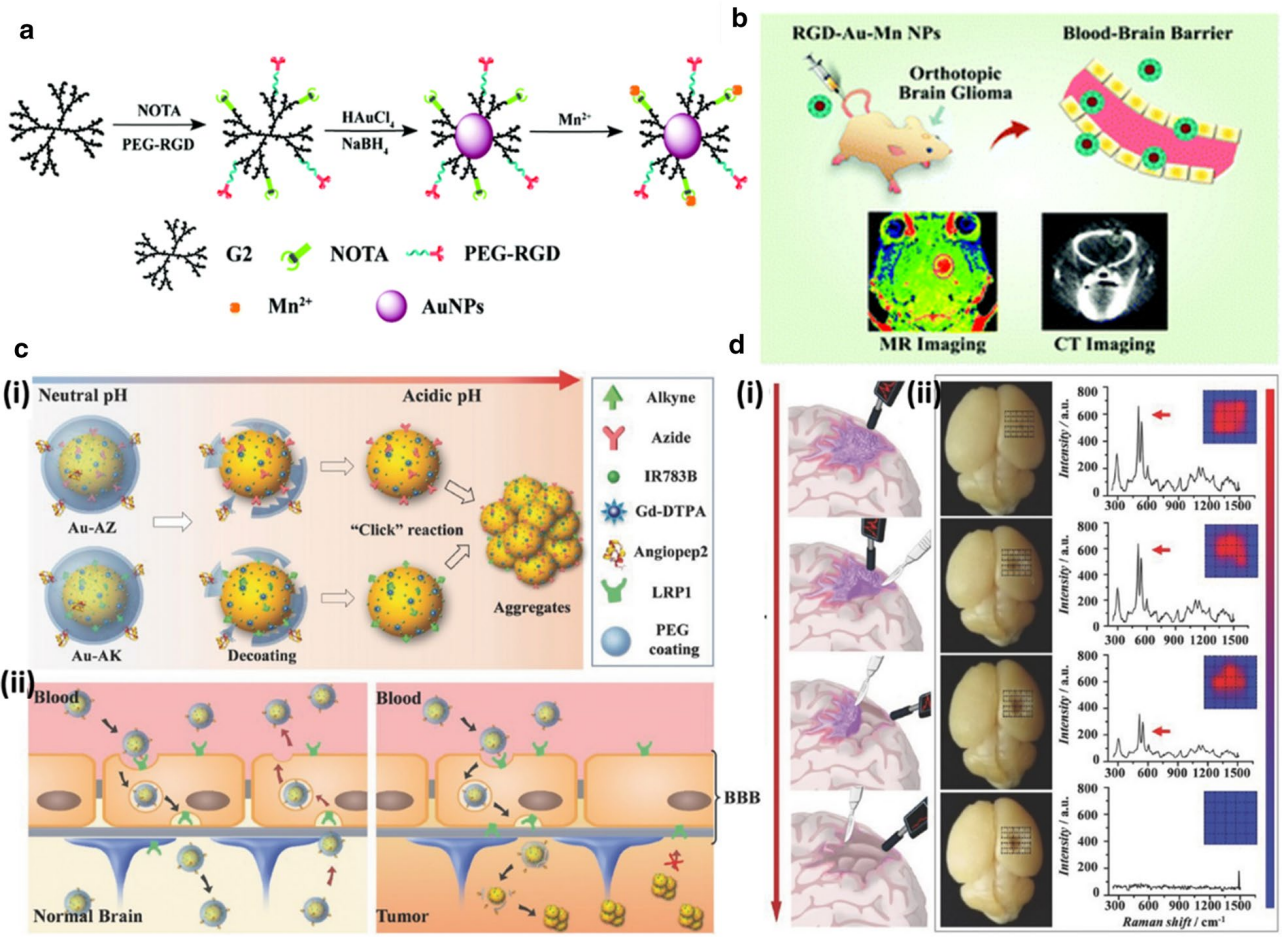
**a** Schematic diagram of the synthesis of RGD-Au–Mn DENPs. **b** Illustration of the MR/CT imaging function of RGD-Au–Mn DENPs. Reprint with permission [195]. Copyright 2019, Royal Society of Chemistry. **c** (i) The cleavage of the PEG coating in physiological acidity triggers aggregation between Au–AZ and Au–AK via “click” cycloaddition reactions. (ii) Due to the LPR1-mediated, bidirectional, BBB-traversing strategy, while the bulky AuNS aggregates are continuously trapped in tumor acidic interstitium, the intact nanoprobes in the normal brain tissue can be transported back into the bloodstream, which increases the sensitivity for the brain-tumor margins. **d** (i) Cartoons illustrating the procedure of Raman-spectroscopy-guided glioma resection. (ii) Photographs of the excised mouse brain bearing U87MG glioma xenografts and Raman spectra at the tumor site during the image-guided tumor resection (Reprint with permission [198]. Copyright 2017, Wiley–VCH)

Computed Tomography (CT) imaging is another commonly used diagnosis method. Since different tissues and organs have varied absorption coefficients towards X-ray, the changes of the X-ray intensity that passes through the body can well reflect the structural alterations of the region of interest. MR imaging has the features of higher spatial resolution, especially for soft tissue like the brain. Unfortunately, because some special tissues or organs of the human body are constantly running while maintaining the basic physiological characteristics of the human body, for example, the non-stop breathing movement of the soft tissue such as lungs will produce motion artifacts during MR scanning, which makes the diagnosis of lung crampons impossible. However, for the diagnosis of lung diseases, the high-density resolution and short scan time of CT imaging solve this problem well. Therefore, due to the particularity of CT imaging in the diagnosis of lung diseases, it is played a vital role in the fight against the Coronavirus disease 2019 (COVID-19) that originated in Wuhan City, Hubei Province of China at the end of December 2019 and spread rapidly around the world, saving many lives [182–192]. Therefore, the combination of MR and CT is capable of better distinguishing the anatomical variations of the lesion areas. Clinically, Omnipaque is the most extensively adopted contrast agent for CT imaging. Like many other functional small molecules for biomedical application, it also suffers from a lack of organ specificity, rapid clearance, poor BBB penetration, and other problems. Thus, developing nanosized contrast agents for MR/CT imaging of glioma is considered a promising strategy [193–195]. Xu et al*.* reported a kind of dendrimer-based nanoprobe for MR/CT imaging of orthotopic glioma [195]. Au nanoparticles were in situ grown into the poly(amidoamine) dendrimers (G2) with the modification of PEG-RGD for targeting and NOTA for Mn^2+^ chelating (Fig. 10a). Notably, the RGD decorated nanoprobe with the hydrodynamic size of 86.6 nm exhibited a higher r_1_ value of 9.88 mM^−1^ s^−1^ than that of the nanoprobe without RGD conjugation (7.94 mM^−1^ s^−1^ with the size of 58.0 nm). This could be ascribed to prolonged rotational correlation time brought by higher hydrophilicity and enlarged molecular volume. Owing to the high atomic number (Z) of Au, effective attenuation of the X-ray was achieved, with the performance even better than commercial Omnipaque. Consequently, the inoculated glioma was displayed only 5 min after intravenous injection and the signal remained distinct for more than 2.5 h (Fig. 10b). Because of the potent absorption of X-ray, Au nanoparticles can also serve as the radiosensitizer for improving the outcomes of radiotherapy, one of the main adjuvant treatments following surgical resection [194].

#### Nanomaterials for other kinds of dual-modal imaging

Surface-enhanced resonance Raman scattering (SERRS) spectroscopy is a kind of Raman spectroscopy of which the signal is greatly enhanced by surface plasmon resonance (SPR). SERRS is an ideal option for guiding tumor resection due to its superb sensitivity, great stability as well as fingerprint-like spectra [196]. Au nanoparticles, apart from the afore-mentioned function like serving as CT contrast agents, can also generate intense SERRS signals through rational design. Thus, the combination of high-resolution MRI for preoperative diagnosis and SERRS for intraoperative guidance represents a promising strategy to treat glioma [197]. Gao et al*.* synthesized a sort of acid-responsive Au nanoprobes for glioma-specific SERRS/MRI [198]. Two kinds of Au nanoparticles, with one kind containing an alkyne group on the surface and the other azide group, were both further coated with Angiopep-2 conjugated PEG shell, which underwent decomposition in acidic conditions. (Fig. 10c) Thereafter, the cycloaddition reaction was initiated between the alkyne group and azide group, resulting in efficient aggregation of the two kinds of Au nanoprobes with the size increasing from 26 to 238 nm after 8 h incubation at pH 6.5. The formation of aggregates largely favored the enhancement of MR signal originating from the pre-anchored Gd-DTPA on the Au surface and SERRS signal. The boosting of longitudinal relaxivity was attributed to the prolongation of the τ_R_ value of the Gd^3+^ chelators, wherein τ_R_ represents the molecular rotational correlation time. As to SERRS, during the formation of aggregates, the interparticle distance decreased, which greatly enhanced the local electromagnetic field and further triggered a strong SERRS signal. After intravenous injection, the nanoprobes effectively traversed BBB and assembled at the glioma region, with the detected bright signal consistent well with the histological H&E staining results. Moreover, the Raman spectroscopic images were also in agreement with the histological analysis, proving the great potential in guiding surgical resection (Fig. 10d).

Nanoprobes for other kinds of dual-modal imaging of glioma, including PET/CT [199, 200], PET/FL [201], PET/Cerenkov luminescence imaging (CLI) [202], PA/FL [203, 204] were also explored by researchers. In a work reported by Cui et al., a porphyrin-based ultrasmall nanostructure was synthesized for PET/FL(NIR) imaging-guided PDT [201]. The fluorescence of porphyrin was quenched in the integrated nanoparticle. Interestingly, after reaching the tumor region, the nanostructure collapsed, with the recovery of fluorescence. Besides, the photodynamic reactivity was also restored. This property benefited tumor-specific FL and PDT. After labeling with ^64^Cu, PET/FL dual imaging was achieved. Yang et al. used albumin, catalase, Au nanorods, and ICG to synthesize a hybrid nanoparticle through a desolvation method [203]. BBB was traversed through albumin-binding protein-mediated transportation and therefore, deep-seated glioma was well recognized through Au nanorods-mediated PA and ICG-mediated FL. Because of the existence of catalase, tumoral H_2_O_2_ could be decomposed to produce O_2_, thus benefiting ICG-mediated PDT in the subcutaneous tumor model. It’s worth noting that MR/PA-involved imaging also accounts for a considerable part in multimodal imaging, which will be introduced in the next chapter as it usually includes other imaging modalities to conduct more complex imaging.

### Nanomaterials for tri-modal or four modal imaging

**Fig. 11:**
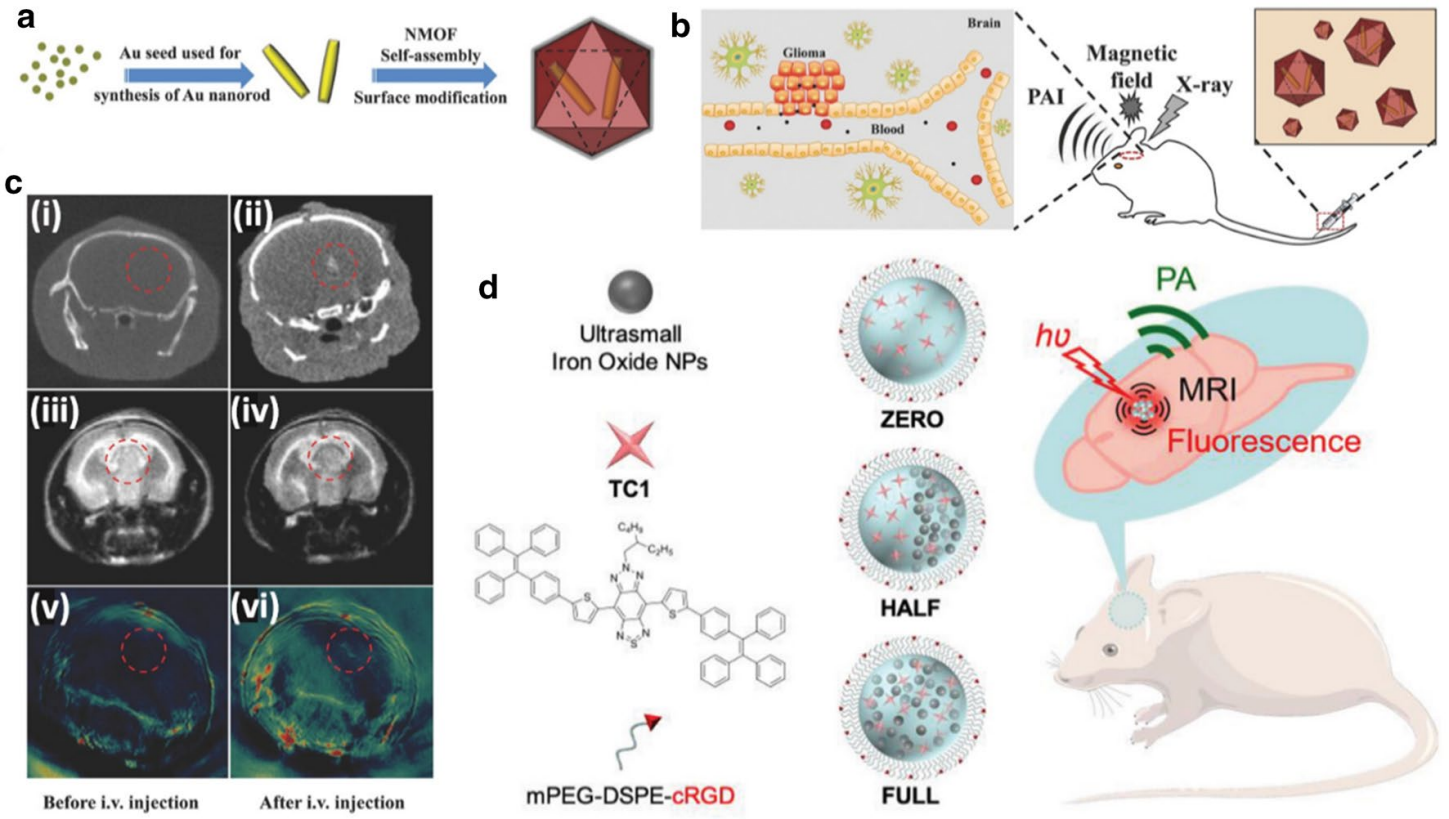
**a** Schematic illustration of the synthesis of Au@MIL-88(Fe) nanostars. **b** Application to multimodality imaging-based tumor diagnosis. **c** (i, ii) CT images of mice before and 12 h after i.v. injection with Au@MIL-88(Fe). (iii, iv) T_2_-weighted MR images of mice before and after i.v. injection with Au@MIL-88(Fe). (v, vi) in vivo PA imaging of tumors in mice before and 12 h after i.v. injection with Au@MIL-88(Fe). Reprint with permission [205]. Copyright 2017, Wiley–VCH. **d** Schematic illustration of the nanostructures of ZERO, HALF, and FULL nanocomposites and multimodal brain tumor imaging applications (Reprint with permission [207]. Copyright 2020, Wiley–VCH)

Since every imaging modality has its strength and weakness, it’s reasonable to integrate as many kinds of imaging modalities as possible into one nanosystem for comprehensive imaging. Nevertheless, due to the limited loading capacity of the nanocarrier and possible conflicts in the imaging mechanism among different modalities like FLI and PAI, it’s impractical to include all of them. How to strike the balance between the performance of each imaging and avoid mutual disruption remains very challenging in the design of a multimodal imaging system. Great efforts have been paid by researchers in this attempt. Kircher et al*.* [205, 206] developed a kind of triple-modality (MR/PA/Raman) imaging nanoprobe for precise diagnosis and intraoperative guidance. Specifically, Au nanosphere with a diameter of 60 nm was firstly coated with a Raman-active layer and then a silica layer of 30 nm. Gd-DOTA molecules were conjugated on the silica shell subsequently. The nanoprobe displayed ultrahigh sensitivity, with 4.88 pM for MRI, 1.22 pM for PAI, and 610 fM for Raman imaging (RI). Due to the highly aggressive nature of glioblastoma, the integrity of BBB could be compromised, which favored the nanoprobe accumulation through the EPR effect. Following intravenous injection, the tumor region could be distinctly observed, with PAI and RI co-registered well with MRI. Moreover, the ex vivo Raman imaging results correlated well with the immunohistochemistry staining outcomes, proving the nanoprobe’s ability to accurately demarcate the tumor margin that benefited the surgical resection. Tian’s group designed a kind of Fe-based MOF (MIL-88(Fe)) encapsulating Au nanorods for MR/CT/PA imaging of the orthotopic glioma (Fig. 11a, b) [205]. Precise growth control of the crystalline MOF outside the Au nanorods was achieved through an ionic/microemulsion method, which preserved the CT/PA imaging ability of the Au nanorods and imparted MRI property (r_2_ = 61.57 mM^−1^ s^−1^ at 1 T magnetic field). At 12 h after intravenous injection, the nanoprobes were effectively accumulated in the glioma site proved by ex vivo Prussian blue staining, generating obvious MR/CT/PA signal for the precise diagnosis (Fig. 11c).

The relative position between all kinds of imaging components and their existing state (monomolecular or aggregated) inside one nanoprobe highly affected the imaging performance. Duan et al*.* reported a sort of nanocomposites mainly composed of synthetic polymer TC1 with a donor–acceptor structure, ultra-small SPIO (USPIO), and mPEG_2000_-DSPE. cRGD was utilized for glioma targeting [207]. Through controlling the water-organic solvent ratio, concentration of each component, and temperature, the distribution of USPIO was accurately regulated, from concentrated in half of the nanosphere (denoted as HALF) to well dispersed in the whole nanosphere (denoted as FULL) (Fig. 11d). Interestingly, the HALF nanoprobes achieved better balance among MR/PA/FL imaging, due to the separation of fluorophore TC1 with USPIO nanoparticles that mitigated the FL quenching effect brought by USPIO. Moreover, the aggregation of TC1 benefited its balance between PA and FL imaging. Consequently, high-efficiency photothermal therapy of orthotopic glioma was achieved under the guidance of tri-modal imaging. Furthermore, four modal imaging was also explored for its possible application in orthotopic glioma models [208]. Song et al*.* synthesized a novel nanoprobe comprised of Fe_3_O_4_ nanoparticle and semiconducting polymer (PCPDTBT) through a nanoprecipitation method. Fascinatingly, the aggregation of Fe_3_O_4_ inside one nanoprobe contributed to the enhancement of transverse relaxation rate (r_2_) and saturation magnetization, with the latter further benefiting magnetic particle imaging (MPI). The existence of semiconducting polymer endowed the nanoprobe with FL and PA imaging ability. Besides, the nanoprobes possessed a long-term blood circulation half-life (49 h), favoring accumulation inside tumor sites. Collectively, four modal imaging of orthotopic glioma was achieved, with excellent tumor contrast to normal tissues.

## Conclusion and prospects

Precise imaging of glioma is vital for efficient treatment. This review summarizes the recent progress of nanomaterials in glioma imaging. Magnetic resonance imaging (MRI), fluorescence imaging (FLI), photoacoustic imaging (PAI), and nuclear medical imaging, as the common and frequently used imaging modalities, are introduced separately with each a brief introduction followed by a detailed summarization of the relevant nanomaterials. Then, nanomaterials for multimodal imaging are introduced, such as MR/FL imaging, MR/CT imaging, MR/FL/PA imaging. Multimodal imaging can provide comprehensive information from different aspects. For example, MRI reflects the anatomical changes of the tumor region while nuclear medical imaging reveals metabolic variations at the molecular level, so the combination of them can simultaneously present structural and functional images, which is very helpful. Also, newly emerged imaging modalities, like chemical exchange saturation transfer (CEST) MRI and surface-enhanced roman resonance spectroscopy (SERRS), are involved in this review. Nanomaterials actively take part in almost all kinds of glioma imaging, demonstrating great potential in efficiently traversing BBB, accurately delineating the glioma margin, and tracing the therapy outcomes.

Although significant progress has been achieved, great challenges still exist before nanomaterials can benefit the patients suffering from glioma: (i) The in vivo stability of nanomaterials needs to be improved. The composition of plasma is very complicated, including various kinds of proteins, high ion concentration. Protein corona is likely to form on the surface of systematically administered nanomaterials, which may cause the loss of targeting ability [43]. Thorough research on the interactions between nanomaterials and plasma is limited, which needs more effort. (ii) For multimodal imaging, further investigation needs to be done to achieve the balance among each kind of imaging. It’s a promising strategy to integrate different kinds of imaging into one nanosystem for a comprehensive diagnosis. However, at present concentrated research on the interactions between different imaging components is limited. An only a better understanding of the underlying mechanisms can lead to the synthesis of multimodal imaging nanoprobes with better performance. (iii) Biosafety-related issues need further eradication. Although tremendous efforts have been put to improve the targeting ability, nanomaterials still tend to be preferentially accumulated in the liver, spleen, or kidney before elimination, which may cause long-term potential toxicity. Besides, the off-targeted nanomaterials dispersed in the brain could cause damage to normal neurons like metal or metal oxide nanoparticles, which needs further systematic evaluation and optimization. The development of nanomaterials for glioma imaging needs interdisciplinary cooperation for its ultimate application in the clinic.

## Data Availability

Not applicable.

## Abbreviations

ABC: ATP-binding cassette
AMT: Adsorption-mediated transcytosis
AMF: Alternating magnetic field
APT: Amide proton transfer
ACQ: Aggregation-caused quenching
AEF: Aggregation-enhanced fluorescence
AIE: Aggregation-induced emission
BBB: Blood–brain-barrier
BSA: Bovine serum albumin
BA: Barbituric acid
Cas: Contrast agents
CT: Computed tomography
CNS: Central nervous system
CED: Convection-enhanced delivery
CPP: Cell-penetrating peptide
CEST: Chemical exchange saturation transfer
CTX: Chlorotoxin
CMT: Carrier-mediated transcytosis
CL: Chemiluminescence
CLI: Cerenkov luminescence imaging
DOX: Doxorubicin
DFT: Density functional theory
ECs: Endothelial cells
EPI: Echo panar imaging
FLI: Fluorescence imaging
FA: Folic acid
FRET: Fluorescence resonance energy transfer
GBM: Glioblastoma
holo-Tf: Holo-transferrin
HA: Hyaluronic acid
HIFU: High intensity focused ultrasound
HSA: Human serum albumin
ICT: Intramolecular charge transfer
ICG: Indocyanine green
LPLNPs: Long persistent luminescence nanoparticles
LED: Light-emitting diode
LIBS: Laser-Induced Breakdown Spectroscopy
LRP: Lipoprotein receptor-related protein
MHT: Magnetic hyperthermia therapy
MT: Magnetization transfer
MMP-2: Matrix metalloproteinase-2
MRI: Magnetic resonance imaging
MOFs: Metal–organic frameworks
MSCs: Mesenchymal stem cells
NK: Natural killer
NOTA: 1,4,7-Triazacyclononane-1,4,7-triacetin acid
NCs: Nanoclusters
PAMAM: 5-Poly(amidoamine)
PEI-PLL: Poly (l-lysine)-grafted polyethyleneimine
PEG-PLA: Poly (ethylene glycol)-poly (lactic acid)
PICs: Polyion complex vehicles
PEG: Poly (ethylene glycol)
PAH: Poly(allylamine hydrochloride)
PAI: Photoacoustic imaging
PTT: Photothermal therapy
PDT: Photodynamic therapy
PET: Positron Emission Tomography
PheoA: Pheophorbide-a
QDs: Quantum dots
RI: Raman imaging
ROS: Reactive oxygen species
RMT: Receptor-mediated transcytosis
SSF: Solid-state fluorescence
SPECT: Single-Photon Emission Computed Tomography
SERRS: Surface-enhanced resonance Raman scattering
SPR: Surface plasmon resonance
SPION: Superparamagnetic iron oxide nanoparticle
Tf: Transferrin
TZM: Temozolomide
TPPTS: Trisodium triphenylphosphine-3,3′,3″-trisulfonate
TPP: Triphenyl phosphonium
uPA: Urokinase plasminogen activator
UCNP: Upconversion nanoparticle
WHO: World Health Organization
